# Identification of a transcriptional signature for the wound healing continuum

**DOI:** 10.1111/wrr.12170

**Published:** 2014-05-20

**Authors:** Matthew A Peake, Mathew Caley, Peter J Giles, Ivan Wall, Stuart Enoch, Lindsay C Davies, David Kipling, David W Thomas, Phil Stephens

**Affiliations:** 1 Wound Biology Group, Cardiff Institute of Tissue Engineering and Repair, Tissue Engineering and Reparative Dentistry, School of Dentistry; 2 Institute of Cancer and Genetics, School of Medicine, Cardiff UniversityCardiff, Wales, United Kingdom; 3 Wound Healing Research Unit, School of Medicine, Cardiff UniversityCardiff, Wales, United Kingdom

## Abstract

There is a spectrum/continuum of adult human wound healing outcomes ranging from the enhanced (nearly scarless) healing observed in oral mucosa to scarring within skin and the nonhealing of chronic skin wounds. Central to these outcomes is the role of the fibroblast. Global gene expression profiling utilizing microarrays is starting to give insight into the role of such cells during the healing process, but no studies to date have produced a gene signature for this wound healing continuum. Microarray analysis of adult oral mucosal fibroblast (OMF), normal skin fibroblast (NF), and chronic wound fibroblast (CWF) at 0 and 6 hours post-serum stimulation was performed. Genes whose expression increases following serum exposure in the order OMF < NF < CWF are candidates for a negative/impaired healing phenotype (the dysfunctional healing group), whereas genes with the converse pattern are potentially associated with a positive/preferential healing phenotype (the enhanced healing group). Sixty-six genes in the enhanced healing group and 38 genes in the dysfunctional healing group were identified. Overrepresentation analysis revealed pathways directly and indirectly associated with wound healing and aging and additional categories associated with differentiation, development, and morphogenesis. Knowledge of this wound healing continuum gene signature may in turn assist in the therapeutic assessment/treatment of a patient's wounds.

The spectrum of wound healing outcomes within an adult human ranges from the enhanced healing observed within the oral mucosa (which has similarities with fetal scarless healing/regeneration) to scarring in normal adult tissues, to nonhealing chronic wounds in the aged. Fibroblasts are central to a successful wound healing response, and alterations in fibroblast functionality across an age-related continuum of wound healing functionality may enhance, facilitate, or prevent normal wound healing and result in differential wound healing processes and outcomes.

It is well established through in vitro investigations that oral mucosal fibroblasts (OMFs) (compared with patient-matched skin fibroblasts) exhibit a fetal-like phenotype.^1^,^2^ At the other end of this continuum is the impaired wound healing phenotype shown by senescent fibroblasts found within chronic (nonhealing) wounds of aged individuals. The response of these cells is impaired^3^,^4^ and in stark contrast to those from the oral mucosa. Interestingly, impaired wound healing is also a feature of certain premature aging conditions, for example, Werner's syndrome.^5^ Between these two extremes of tissue response, the normal wound healing response, as found in adult dermal tissues, features an intermediate rate of wound repair, which results ultimately in a repair of the tissue but inevitably the production of scar tissue (Figure 1).

**Figure 1:**
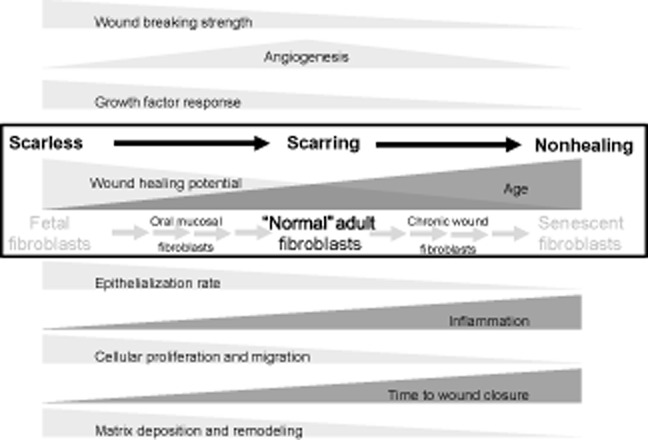
An overview of the cellular and clinical characteristics of the functional wound healing continuum. Key processes associated with scarless, scarring, and nonhealing wound repair are highlighted as they increase and decline across the repair spectrum. Critical to this is the role of the fibroblast and the “aging” transition from fetal fibroblasts, through adult fibroblasts to senescent fibroblasts.

The use of microarray technologies to carry out genome-wide analysis of gene expression has been used to produce gene signatures for skin from young and old individuals^6^ and expression profiles of several different cell types isolated from healthy skin.^7^ This technology has also been utilized to compare skin cells (fibroblasts) from multiple anatomical locations.^8^ Analysis of both acute^9^ and chronic wound gene signatures has also been undertaken.^6^,^10^,^11^ Initial analysis of the transcriptional response of cultured human fibroblasts to serum showed that it mirrors many aspects of the normal wound healing response.^12^ Analysis of this wound healing/serum response in fibroblasts isolated from multiple anatomical sites leads to the description of a “core/common serum response” signature of genes whose differential expression pattern was shared.^13^ Research has started to identify gene expression profiles associated with wound healing through in vitro^4^,^14^,^15^ and in vivo^10^,^11^ studies. Such information is beginning to generate insight into the wound healing process, to identify potential prognostic indicators^16^ and also to aid clinical decision making.^10^

In this study, we investigated the presence/absence of a pattern of gene expression that may correlate to the “continuum” of wound healing phenotypes observed, spanning enhanced (adult oral mucosa), normal (adult skin), and impaired (adult chronic venous leg ulcers) wound healing in order to get insight into the genes and processes involved. In the future, this (wound healing-related) expression information and the markers identified in this way may be helpful in assessing the “status” of healing within a clinical wound and to custom develop appropriate treatment regimes.

## MATERIALS AND METHODS

### Patients and tissues

Fibroblasts (*n* = 4) were established from either oral (buccal) mucosa (OMF) and patient-matched skin from the head/neck region (normal skin fibroblast [NF]1) or chronic venous leg ulcer wound tissues (chronic wound fibroblast [CWF]) and patient-matched “normal” skin (NF2) cultured from the ipsilateral thigh, respectively (see Supporting Information Table S1 for tissue details). All tissue samples (6 mm biopsy) were obtained after South East Wales Research Ethics Committee approval and written informed patient consent either from patients attending for minor oral surgery at the School of Dentistry, Cardiff, United Kingdom or from patients attending the Wound Healing Clinic at the University Hospital of Wales, Cardiff, United Kingdom. Patients with diabetes, systemic immunosuppression, or evidence of local infection were excluded from the study.

### Establishment of oral mucosa, chronic wound, and patient-matched normal fibroblasts

Cultures were established by a single-cell suspension technique following enzymatic degradation of the specimens as previously described.^1^ The population doublings (PDs) of the cell populations (*n* = 4 OMF/NF1 and *n* = 4 CWF/NF2) in vitro were derived from direct counting of cell numbers at each passage.

### Affymetrix gene expression microarray analysis

RNA was extracted from serum-stimulated cells as described.^12^ Briefly, cells (passage 5 or 6) were seeded at a density of 1 × 10^4^ cells/cm^2^ in 10 cm diameter dishes and cultured for 24 hours. Cells were then serum starved for 48 hours to induce quiescence. Cells were then restimulated with serum and harvested at 0 and 6 hours (the time point where maximal fibroblast response has been previously shown following serum activation^12^) and RNA was extracted. RNA concentration was quantified on a Genequant pro spectrophotometer (GE Healthcare Life Science, Little Chalfont, Bucks, United Kingdom) by measuring the absorbance at 260 nm. RNA integrity was determined using an RNA nanoLabChip kit on an Agilent 2100 bioanalyzer (Agilent Technologies, Workingham, Berks, United Kingdom). Samples (*n* = 4 OMF/NF1 and *n* = 4 CWF/NF2) were labeled and hybridized to Affymetrix GeneChips (U133A) by the Central Biotechnology Service, Cardiff University. After scanning and probe-level quality control assessment, expression summary values were extracted using the MAS 5.0 algorithm. Expression of a number of the genes assessed using these GeneChips was corroborated by quantitative polymerase chain reaction (Supporting Information Figure S1). A single GeneChip was used for each experimental condition. However, the data for the 6-hour sample in skin of one patient was found to be an outlier during probe-level quality control analysis, and thus the 0-hour and 6-hour data from this sample was excluded from all further analysis. Thus, for subsequent continuum analysis, a 30-GeneChip dataset was used that comprised the 0-hour and 6-hour expression data from the four OMF samples and four CWF samples, together with the 0-hour and 6-hour data from their patient-matched normal fibroblasts (NF1 and NF2).

### Continuum analysis

**Figure 2:**
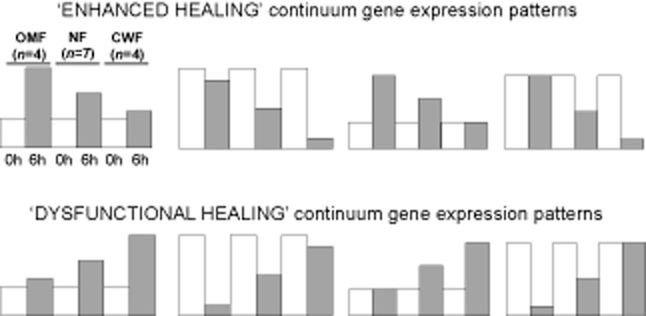
The “continuum” patterns of gene expression investigated. A sampling was noted as having a “continuum pattern” if there was a minimum twofold change in expression level in response to serum in at least the normal (middle) and one of the “ends” of the continuum, and a correlation coefficient of 0.7 or greater to either a positive or negative continuum gradient. The continuum gradient was defined as either increasing (OMF < NF < CWF) or decreasing (OMF > NF > CWF). CWF, chronic wound fibroblast; NF, normal skin fibroblast; OMF, oral mucosal fibroblast.

The aim of the continuum analysis was to identify serum responsive genes whose expression were found to be either greatest in OMF and decreased in NF1 and NF2 to CWF, or conversely were lowest in OMF and then showed increased levels in NF1 and NF2 to CWF. Each of the ∼22,000 probe sets was analyzed individually. For each probe set, all 112 possible subsets were selected that comprised the matched 0-hour and 6-hour data for one OMF, one NF, and one CWF sample. Each subset was then assessed for its “compliance/fit” with a series of hypothetical gene profiles reflective of either enhanced wound healing (Figure 2, top) or dysfunctional wound healing (Figure 2, bottom). A subset was scored as having a “continuum pattern” if it matched the following criteria. First, there was a minimum of a twofold change in expression level in response to serum in the NF pair and either or both of the CWF and OMF. Second, a correlation coefficient of 0.7 or greater to one of the hypothetical profiles was required. The direction of the continuum gradient was defined as either increasing (OMF < NF < CWF) or decreasing (OMF > NF > CWF). For each probe set, the number of the 112 subsets where the data were scored as matching the continuum was recorded. Statistical probability was then determined by a permutation-based test where 10,000 random subsets were created for each probe set, each made by selecting three random 0-hour/6-hour pairs but ignoring the tissue type. For each probe set, the number of times it was scored as a continuum gene in the 112 subsets was then compared with the number of times it was scored as a continuum gene in the 10,000 random subsets. A *p*-value was then generated using a chi-square test. Probe sets with a false discovery rate (FDR) corrected *p*-value of <0.05 were selected. These probes were then annotated and analyzed by overrepresentation analysis using the Database for Annotation, Visualization and Integrated Discovery (DAVID) version 6.7 (http://www.david.abcc.ncifcrf.gov). Gene Ontology (GO) biological process (GOTERM_BP_ALL) categories that were identified (*p* < 0.05) as overrepresented were then ranked by Expression Analysis of Systematic Explorer (EASE) score. Probe sets were then classified as having an ascending or descending pattern based on being in a particular group when at least one quarter of the samplings matched for one or more of the continuums (continuum patterns as laid out in Figure 2), resulting in the final enhanced healing and dysfunctional healing gene sets.

## RESULTS

### Fibroblasts show a continuum of proliferative potential

**Figure 3:**
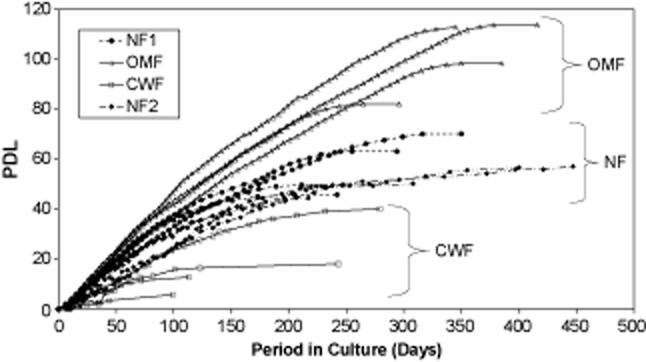
The growth potential of fibroblasts isolated from adult oral mucosal, adult skin, and adult chronic venous leg ulcer tissues (*n* = 4 OMF; *n* = 7 NF1/NF2; *n* = 4 CWF). CWF, chronic wound fibroblast; NF, normal skin fibroblast; OMF, oral mucosal fibroblast; PDL, population doubling level.

To illustrate the concept of a continuum of cellular responses between OMF, NF, and CWF, the growth kinetics of these cells were investigated through senescence. OMF had the greatest proliferative potential, proliferating for much longer in culture and senescing later than patient-matched NF1 (mean of 101.5 PD vs. 56.8 PD, respectively; *p* < 0.02; Figure 3). Conversely, CWF had the least proliferative potential, growing for a much shorter period of time in culture and senescing earlier than patient-matched NF2 (mean of 18.04 PD vs. 48.03 PD, respectively; *p* < 0.02). A comparison of NF1 (head/neck) with NF2 (thigh) showed a similar growth potential (*p* > 0.2). Therefore, for further analysis, three “sets” will be considered: OMF (four patient samples), NF (seven patient samples; 3× head/neck [one of the four samples failed quality control, see above] and 4× thigh), and CWF (four patient samples).

### Defining a gene signature for differential wound healing

**Figure 4:**
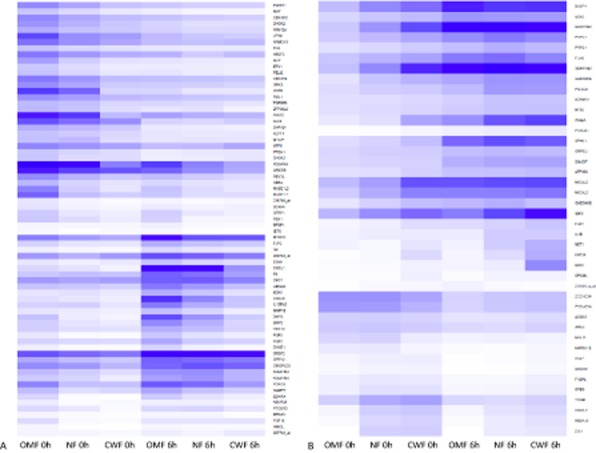
Expression heat map of “enhanced healing” (A) and “dysfunctional healing” (B) groups of genes arranged to highlight the gradient of expression from top to bottom of the continuum at 0 hour and 6 hours time points. This heat map was generated by mapping the data onto a light-dark range representing low–high expression levels, respectively. CWF, chronic wound fibroblast; NF, normal skin fibroblast; OMF, oral mucosal fibroblast.

**Figure 5:**
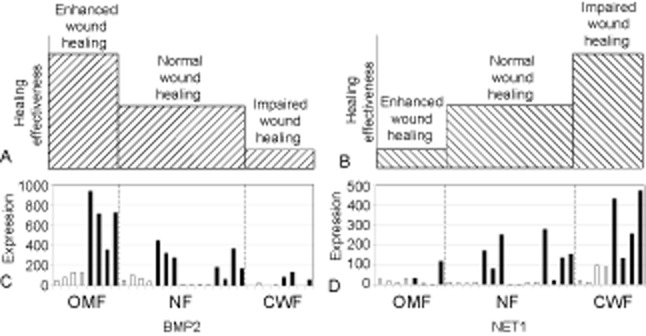
Pattern of expression of genes present within the “enhanced healing” (A) and “dysfunctional healing” (B) groups. Examples of relative gene expression from each of these two groups are shown (C & D respectively; open bars = 0-hour samples, closed bars = 6-hour samples). Bone morphogenetic protein 2 (*BMP2*) and neuroepithelial cell transforming gene 1 (*NET1*) were chosen to represent examples of (1) a gene linked to enhanced/preferential healing (i.e., *BMP2* stimulated significantly in OMF [scarless] but not CWF [nonhealing]); and (2) a gene linked to dysfunctional/chronic healing (i.e., *NET1* stimulated in CWF [nonhealing] but not OMF [scarless]). CWF, chronic wound fibroblast; NF, normal skin fibroblast; OMF, oral mucosal fibroblast.

We used a permutation-based test to identify probe sets that were differentially expressed in OMF, NF, and CWF in a pattern consist with a possible role in a wound healing continuum (i.e., those demonstrating an increasing [OMF < NF < CWF] or decreasing [OMF > NF > CWF] expression gradient from one “end” of the continuum to the other). For the differential fibroblast populations, all possible sampling of one OMF sample, one NF sample, and one CWF sample (giving the 112 different samplings of the dataset) were assessed for correlation of this subset to the theoretical profiles of a healing continuum (Supporting Information Figure S2). A total of 112 probe sets displayed patterns of expression consistent with that of an ideal continuum (Supporting Information Figure S3; FDR controlled *p* < 0.05) and were further subdivided into ascending or descending continuum patterns. The largest group of genes identified by this continuum identification analysis was the enhanced healing group (Figure 4A and Supporting Information Table S2), which showed a descending pattern of expression across the continuum. Sixty-six genes were present within this group, and although they have diverse functions, they do have the common characteristic of having their highest expression in the fetal-like (scarless) oral mucosa and decreasing to their lowest levels in the impaired nonhealing phenotype of the chronic wound (example shown in Figure 5A and C). Thirty-eight genes were present within the dysfunctional healing group (Figure 4B and Supporting Information Table S3) and were at low levels in cells where healing was enhanced and were at increased levels in cells associated with poor wound healing capacities (example shown in Figure 5B and D). One gene (*MYLIP*) showed a more complex pattern of expression and fell into more than one of the gene expression pattern classifications.

### Functional analysis of the gene signature

Overrepresentation analysis was carried out on the continuum gene set using GO and DAVID EASE to identify functional classifications that were enriched. Within the complete continuum gene set, several functional categories were overrepresented, including a large representation for the role for development/differentiation and their regulation. Furthermore, several categories of relevance to wound healing and aging (Supporting Information Table S4) were also identified including “regulation of cell proliferation,” which was the most significantly overrepresented category in this gene set. Overrepresentation of the “response to wounding” and “wound healing” functional pathways in addition to other wound healing-related categories including cell migration and its regulation, growth factor signalling pathways, angiogenesis, cell–cell signalling, categories associated with response to stress and extracellular stimuli, BMP signalling pathways, apoptosis, and the positive regulation of gene expression were all shown. Of specific interest with respect to tissue repair, chemokine ligand (*CXCL*)-1 and 6, platelet-derived growth factor receptor, alpha polypeptide (*PDGFRA*), fibroblast growth factor (*FGF*)-5 and 18, coagulation factor III, and BMP2 were identified within the enhanced wound healing gene set (Supporting Information Table S2), whereas the wound healing-related genes *SERPINE1*, *FGF1*, and interleukin-1 beta (*IL-1β*) were shown within the dysfunctional wound healing gene set (Supporting Information Table S3).

## DISCUSSION

This study has identified group of continuum genes that were further subdivided with respect to which particular pattern of continuum-like expression they display, namely, those which show “descending” or “ascending” patterns of expression across the continuum. Those genes whose expression diminished across the continuum (i.e., OMF > NF > CWF) may be associated with a positive, enhanced, or effective wound healing phenotype (which will be referred to hereafter as the *enhanced healing* group). Alternatively, those genes whose expression increased across the same continuum were likely to be associated with a negative or impaired wound healing phenotype (which will be referred to hereafter as the *dysfunctional healing* group). Theoretically, the analysis could have been undertaken utilizing a different sequence, but the above one was specifically chosen to link to the distinctly different wound healing potentials of the source tissues from which the cells (with such distinct phenotypes) were sourced.

The expression analysis presented here has shown that genes associated with a number of processes including key components of the wound healing response are differentially regulated by serum (a surrogate wound healing stimulus) in a manner which varies depending upon the source of fibroblasts being studied. The wound healing capacities of these different fibroblast populations can be matched to the “top,” “middle,” and “bottom” of the age-related wound healing continuum, and a number of genes have shown patterns of differential expression, which are associated with either a decreasing (enhanced wound healing) or an increasing (dysfunctional wound healing) continuum pattern. Other processes, less directly linked to wound healing, were also found to correspond to this continuum pattern, specifically those relating to system and organ development, developmental regulation, and morphogenesis. This therefore is further confirmation of the previous reported similarities between some elements of the tissue repair and tissue development pathways.

Age-related biological effects in impaired/delayed wound healing have been shown when comparing aged vs. young individuals in both human and animal models.^17^,^18^ This “age-related” differential capacity for wound healing is also found in cells (e.g., fibroblasts) isolated from different locations on the same individual.^8^,^10^,^15^ Within this study, several characteristics of these different cell populations (OMF, NF, and CWF) mirror those seen across an aging healing continuum (most significantly their differential capacity for effective wound healing and their observed differences in proliferative potential). The replicative capacity of fibroblasts has been shown to reduce with age, which is associated with a reduction in growth factor responsiveness^19^ and the increased production of markers of replicative senescence.^20^

Effective wound healing depends upon the action and interaction of thousands of genes taking part in a number of key biological processes. A number of the genes present within the described *continuum wound healing profile* have been previously noted to alter expression in different biological wound models and in an age-related manner. Such observations validate our approach and suggest that it is relevant to dissect some aspects of the continuum of wound healing. These and other genes in the profile are associated with pathways and processes linked to determining the effectiveness of the wound healing response (e.g., genes associated with cell proliferation and growth factor pathways, cell migration, cell signaling, chemotaxis, in addition to genes functionally associated with wound healing).

Of the enhanced healing genes identified by this analysis, CXC chemokines CXCL1 and CXCL6 are of particular interest in wound healing biology due the crucial role of inflammation during the process. CXCL1 is thought to initiate neutrophil recruitment immediately after injury.^21^ Reduced induction of these CXC chemokines within chronic wounds may be indicative of a reduced ability to induce an acute inflammatory response. Gene coagulation factor III is also associated with the inflammatory phase of the wound healing response, and increased expression is known to result in enhanced wound healing.^22^ Reducing levels of enhanced gene PDGFRA expression, across the continuum may partially explain these previously noted reductions in responsiveness to PDGF observed with increased aging^23^ and in cells isolated from chronic wounds.^24^

Of those wound healing genes found within the “dysfunctional” continuum signature, the proinflammatory cytokine IL-1β is known to mediate important functions in the early and late courses of inflammation, trauma, and wound healing.^25^ *SERPINE1* (plasminogen activator inhibitor-1 [PAI-1]) expression has been noted to increase with age^26^ and is an essential mediator of replicative senescence.^27^ PAI-1 is also known to be a key component of the transcriptional response to wounding in epithelial cells.^28^

The study of gene expression at a genome-wide level can be utilized to gain insight into specific biological processes and diseases. Among the most heavily examined examples of such studies are those targeted at cancer. In this field, transcriptional profiling has led to the identification of gene expression signatures for classification of disease stage/state, clinical outcome, and response to therapy.^29^–^31^ Indeed, a serum-induced fibroblast gene signature has been used as a predictive indicator for clinical outcome prediction in multiple types of cancer.^13^

However, microarray analysis of (human) skin and wound healing has yet to reach this point of application. Microarray technology has been utilized to examine normal and wounded human skin across a number of patients,^11^,^32^,^33^ and expression in healthy and wounded skin has also been compared with that of in vitro models.^34^ Studies have also compared the expression profiles of healing and scarring tissues of both humans and multiple animal models.^35^ Analysis of human oral mucosal wound healing expression profiles^36^ has been complemented by comparisons of the gene expression profiles of similar (scarless) oral mucosal wounds and (scarring) skin wounds of pigs and humans.^37^ The expression profiles of fetal skin wounds in the early (scarless healing) and late (scar forming) gestational periods of rats were also compared.^38^ Microarray-based gene expression analysis of a time series of the acute wound healing response has also been undertaken in rats.^39^ Early in vitro analysis of the transcriptional response of human fibroblasts to serum showed that it mirrored many aspects of the normal wound healing response.^12^ Comparison of fetal and adult fibroblasts from multiple anatomical sites indicated that gene expression differed both following serum starvation and also in response to serum stimulation.^8^ Among those genes was fibroblast growth factor 18, a gene which is part of the enhanced healing profile described here. Similarly, while all the genes were not in common, distinct similarities were observed between the functional categories overrepresented in this study with those reported by others in fibroblasts in response to serum (e.g., cell motility, extracellular matrix remodeling, cell–cell signaling, cell proliferation, and angiogenesis^8^).

A gene profile for nonhealing venous leg ulcers has been reported based on tissue samples taken from the wound bed and wound edge of patients with healing and nonhealing chronic venous leg ulcers.^11^ These investigators suggested that such an approach may have implications for the development of novel therapies and prognostic indicators for wound healing. Further comparisons of gene profiles from chronic venous leg ulcers and healthy, unwounded skin showed that differentiation and activation markers were deregulated in the nonhealing samples.^11^ In another investigation, Brem et al.^10^ determined that the nonhealing edge of venous leg ulcers and the corresponding adjacent normal skin also had distinct and reproducible gene expression signatures and that fibroblasts isolated from these two sites differed in morphology and capacity to repair wounds in support of our own findings.^4^

These investigations have, for the first time, identified a novel gene signature for differential wound healing across the entire spectrum of the repair process. A profile of 104 genes has been identified, some of which have previously identified functions in wound healing and some of which appear to be novel in this area. This analysis gives insight into some of the genes that may be involved in (and possibly determine) the effectiveness of the wound healing process in a particular acute or chronic wound environment. Further analysis of the expression of some/all of these signature genes may assist in the determination of the healing potential of the cells/sample/patient under investigation. While the purpose of this investigation was to specifically assess gene expression in relation to wound healing outcome (which technologically could be commercially translatable), future analysis of protein data and investigation within the tissues themselves to confirm such functional wound healing difference would add a further, interesting dimension to this analysis. A further potential therapeutic value of this signature will be in the assessment of the effectiveness of a wound healing therapy through monitoring changes in the expression of those genes over time in patients.

## Abbreviations

CWF: Chronic wound fibroblast
CXCL: Chemokine ligand
DAVID: Database for Annotation, Visualization and Integrated Discovery
EASE: Expression Analysis of Systematic Explorer
ECM: Extracellular matrix
FDR: False discovery rate
FGF: Fibroblast growth factor
GO: Gene Ontology
IL-1ß: Interleukin-1 beta
NF: Normal skin fibroblast
OMF: Oral mucosal fibroblast
PAI: Plasminogen activator inhibitor
PD: Population doubling
PDGF: Platelet-derived growth factor
PDGFRA: Platelet-derived growth factor receptor, alpha polypeptide

## Supporting Information

**Figure S1.** Gene-matched quantitative polymerase chain reaction (qPCR) data and Affymetrix (Affy) microarray data from *n* = 3 independent patients for a number of genes demonstrating the validity and robustness of the microarray data (note the similar expression profiles across the different samples [NF and CWF] and time points [0 and 6 hours]). Values = mean (*n* = 3) ± standard error of the mean (SEM). CWF, chronic wound fibroblast; CXCL, chemokine ligand; NF, normal skin fibroblast. **Figure S2.** Schematic of the continuum analysis methodology. One hundred twelve samplings were possible (four OMF × seven NF1/NF2 × four CWF) with each assessed for its “compliance/fit” with a wound healing continuum. To account for the possibility of a probe set following the desired expression pattern by chance, data were also randomly sampled. CWF, chronic wound fibroblast; NF, normal skin fibroblast; OMF, oral mucosal fibroblast. **Figure S3.** Expression heat map of continuum-like genes. **Table S1.** Tissue details for each OMF, NF, and CWF cell strain. **Table S2.** A list of genes expressed during enhanced wound healing obtained from analysis of the microarray data. **Table S3.** A list of genes expressed during dysfunctional wound healing obtained from analysis of the microarray data. **Table S4.** Functional classification of wound healing continuum genes.
