# Supplementary material for: Identification of a transcriptional signature for the wound healing continuum
Source: Wound Repair Regen. 2014 May 20;22(3):399–405. doi: 10.1111/wrr.12170 (PMC4230470; doi:10.1111/wrr.12170)
Supplement: Supplementary file 1 [file wrr0022-0399-SD1.pdf]

Figure 1S

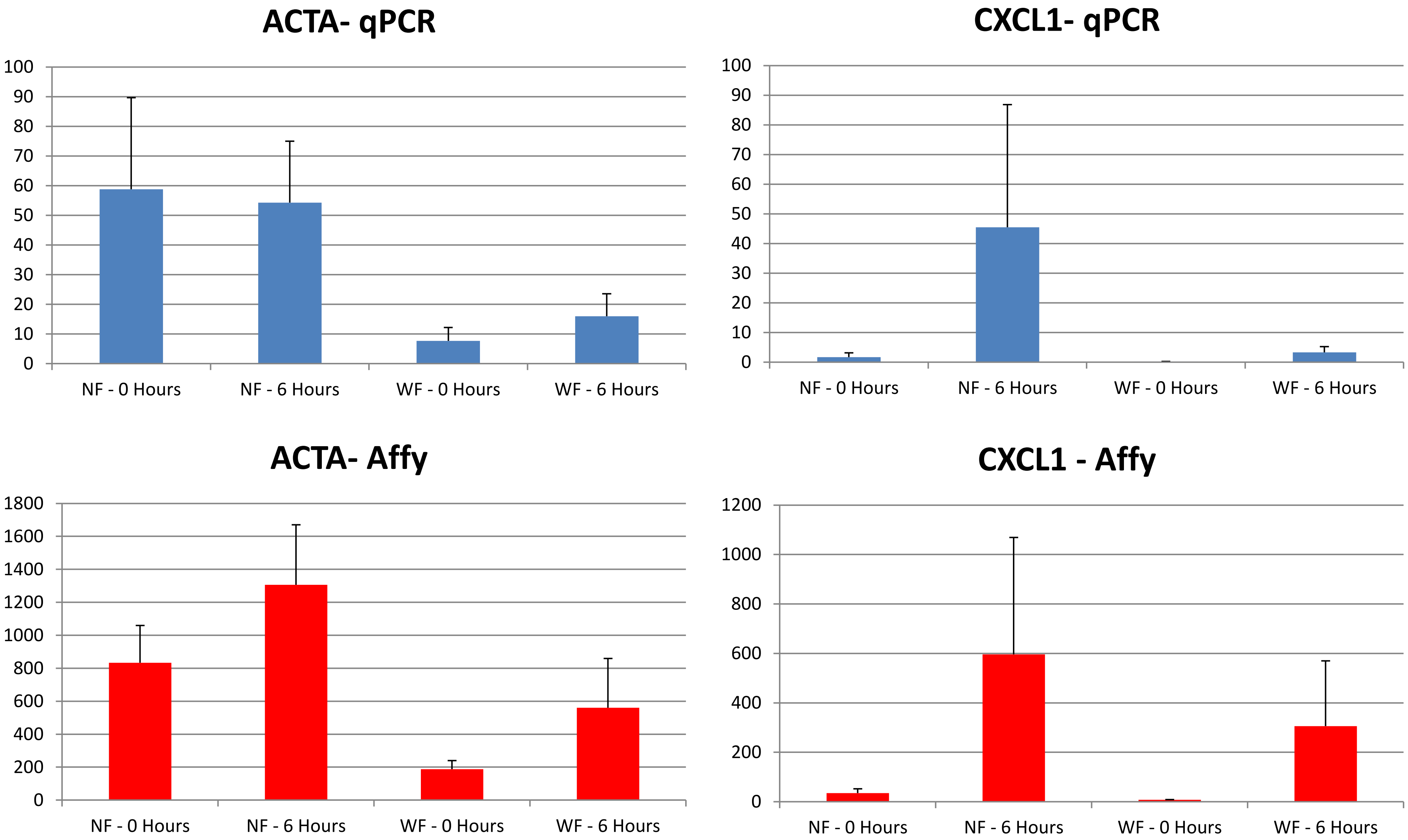
ACTA- qPCR
100
90
80
70
60
50
40
30
20
10
0
NF - 0 Hours
NF - 6 Hours
WF - 0 Hours
WF - 6 Hours
CXCL1- qPCR
100
90
80
70
60
50
40
30
20
10
0
NF - 0 Hours
NF - 6 Hours
WF - 0 Hours
WF - 6 Hours
ACTA- Affy
1800
1600
1400
1200
1000
800
600
400
200
0
NF - 0 Hours
NF - 6 Hours
WF - 0 Hours
WF - 6 Hours
CXCL1 - Affy
1200
1000
800
600
400
200
0
NF - 0 Hours
NF - 6 Hours
WF - 0 Hours
WF - 6 Hours

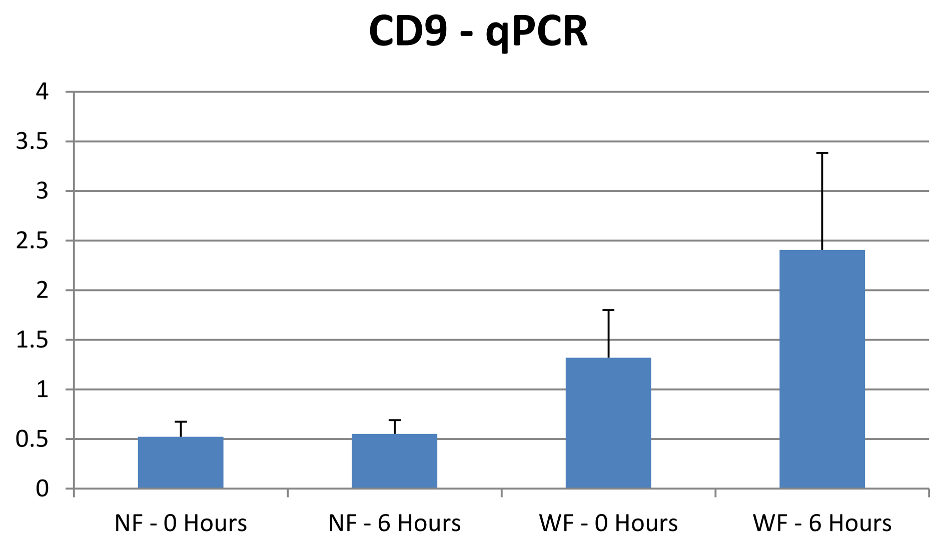
CD9 - qPCR
4
3.5
3
2.5
2
1.5
1
0.5
0
NF - 0 Hours
NF - 6 Hours
WF - 0 Hours
WF - 6 Hours

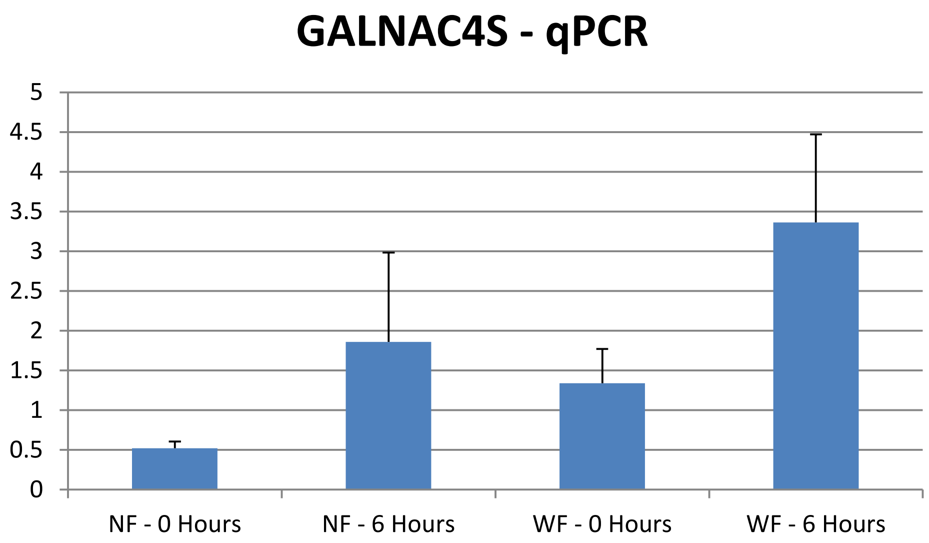
GALNAC4S - qPCR
5
4.5
4
3.5
3
2.5
2
1.5
1
0.5
0
NF - 0 Hours
NF - 6 Hours
WF - 0 Hours
WF - 6 Hours

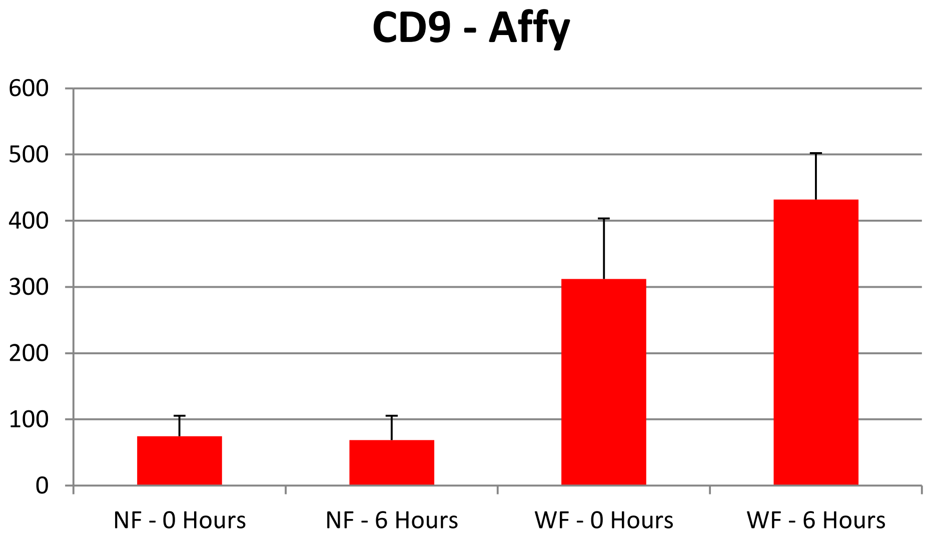
CD9 - Affy
600
500
400
300
200
100
0
NF - 0 Hours
NF - 6 Hours
WF - 0 Hours
WF - 6 Hours

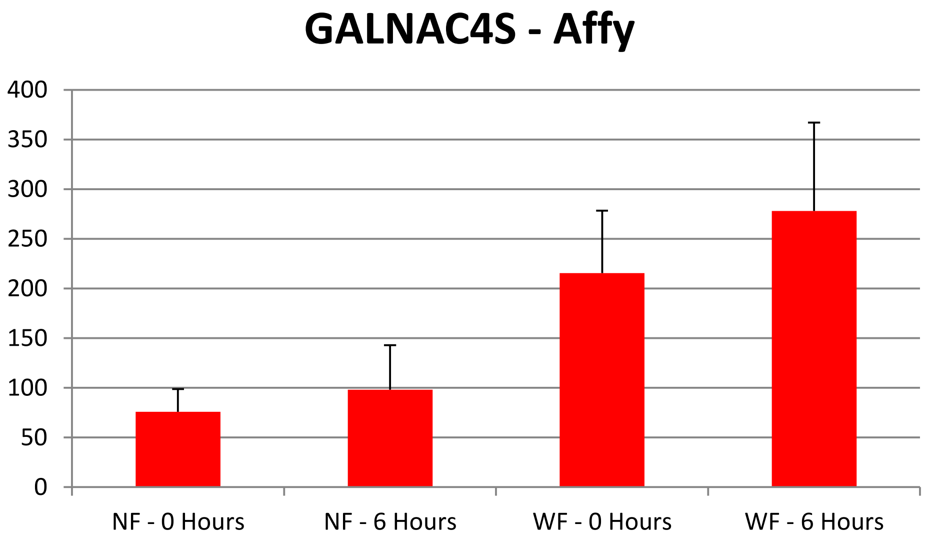
GALNAC4S - Affy
400
350
300
250
200
150
100
50
0
NF - 0 Hours
NF - 6 Hours
WF - 0 Hours
WF - 6 Hours

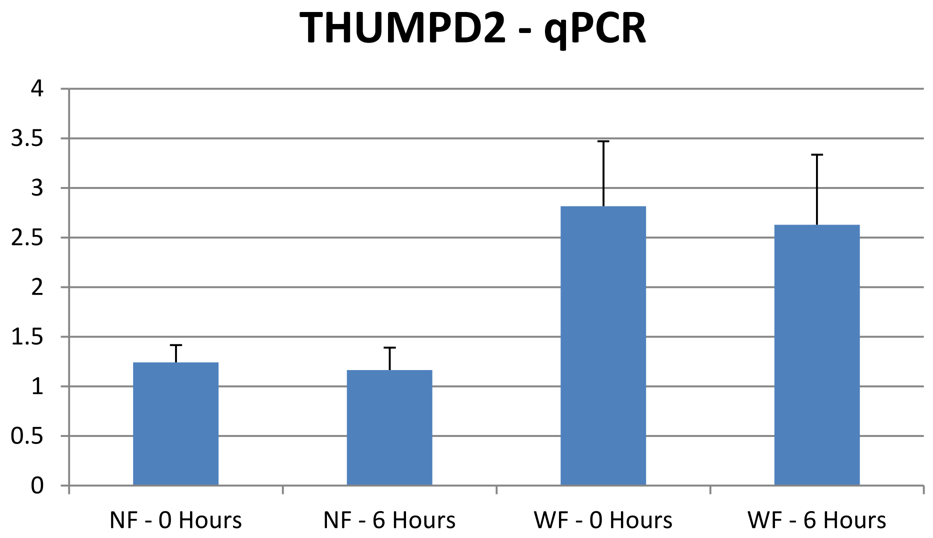
THUMPD2 - qPCR
4
3.5
3
2.5
2
1.5
1
0.5
0
NF - 0 Hours
NF - 6 Hours
WF - 0 Hours
WF - 6 Hours

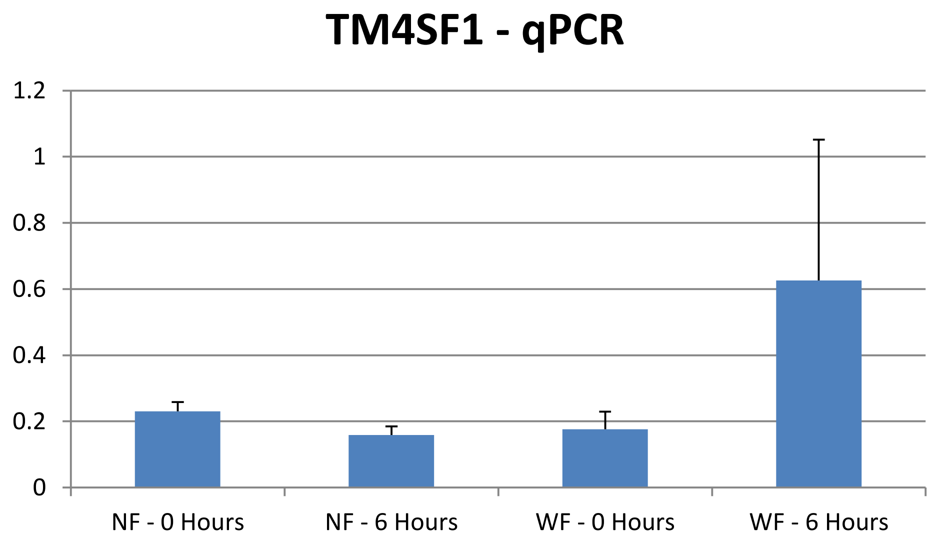
TM4SF1 - qPCR
1.2
1
0.8
0.6
0.4
0.2
0
NF - 0 Hours
NF - 6 Hours
WF - 0 Hours
WF - 6 Hours

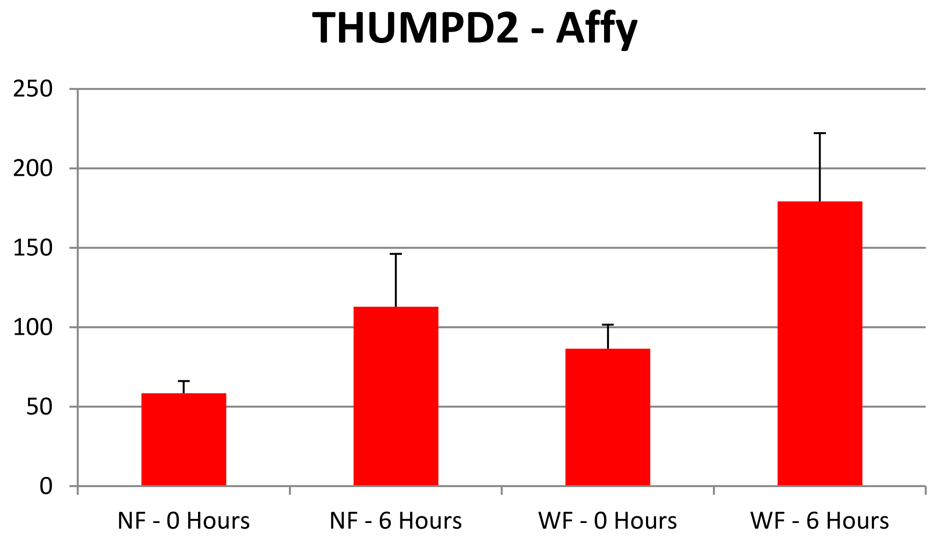
THUMPD2 - Affy
250
200
150
100
50
0
NF - 0 Hours
NF - 6 Hours
WF - 0 Hours
WF - 6 Hours

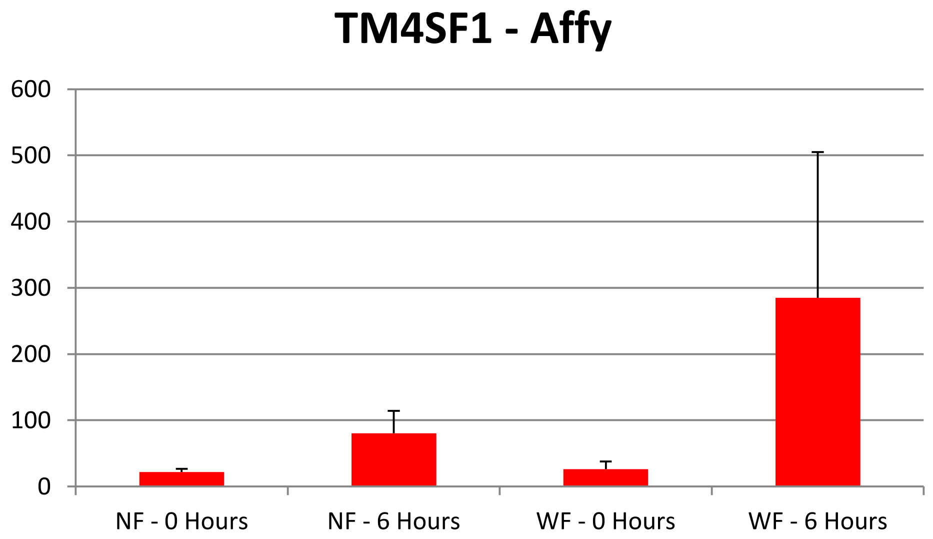
TM4SF1 - Affy
600
500
400
300
200
100
0
NF - 0 Hours
NF - 6 Hours
WF - 0 Hours
WF - 6 Hours

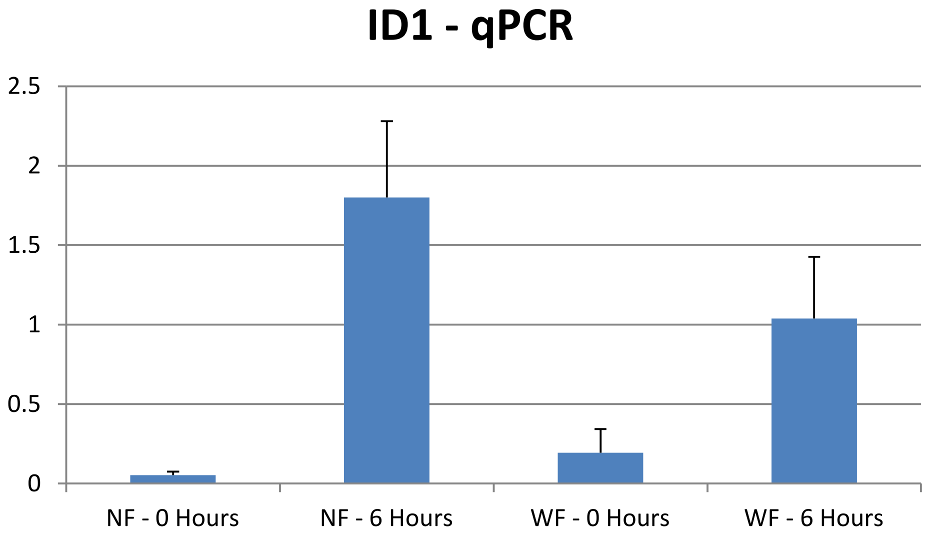
ID1 - qPCR
2.5
2
1.5
1
0.5
0
NF - 0 Hours
NF - 6 Hours
WF - 0 Hours
WF - 6 Hours

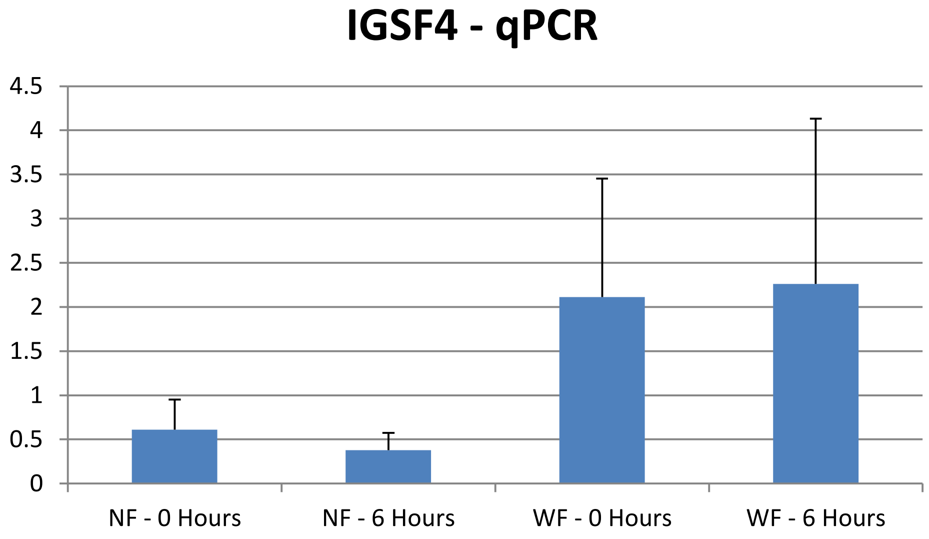
IGSF4 - qPCR
4.5
4
3.5
3
2.5
2
1.5
1
0.5
0
NF - 0 Hours
NF - 6 Hours
WF - 0 Hours
WF - 6 Hours

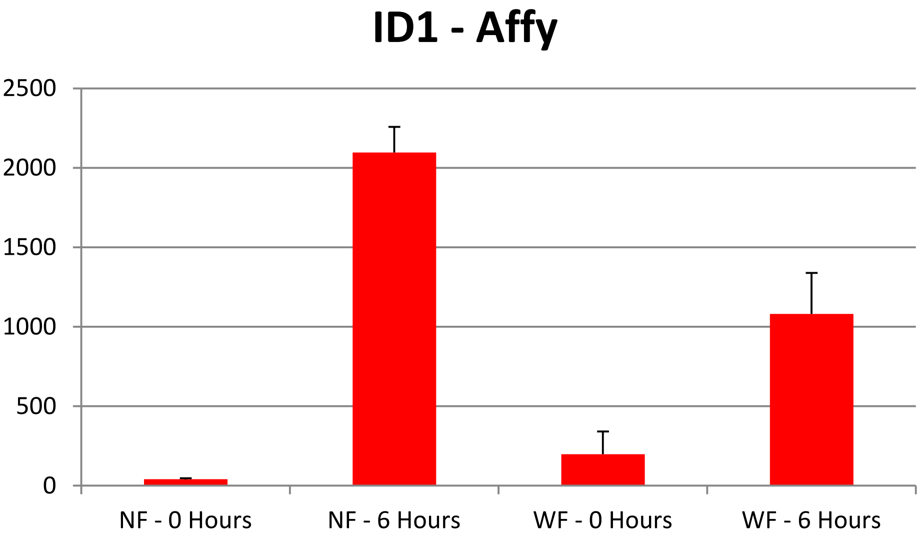
ID1 - Affy
2500
2000
1500
1000
500
0
NF - 0 Hours
NF - 6 Hours
WF - 0 Hours
WF - 6 Hours

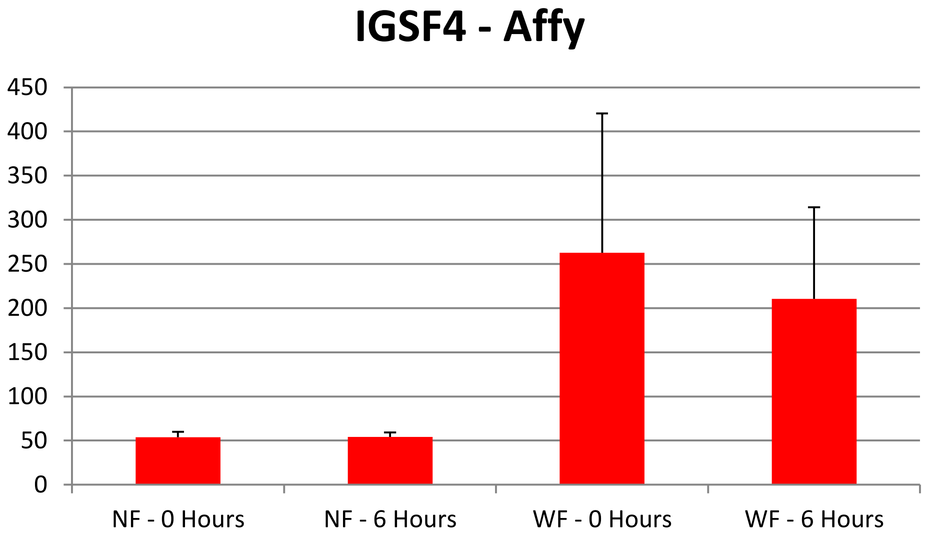
IGSF4 - Affy
450
400
350
300
250
200
150
100
50
0
NF - 0 Hours
NF - 6 Hours
WF - 0 Hours
WF - 6 Hours

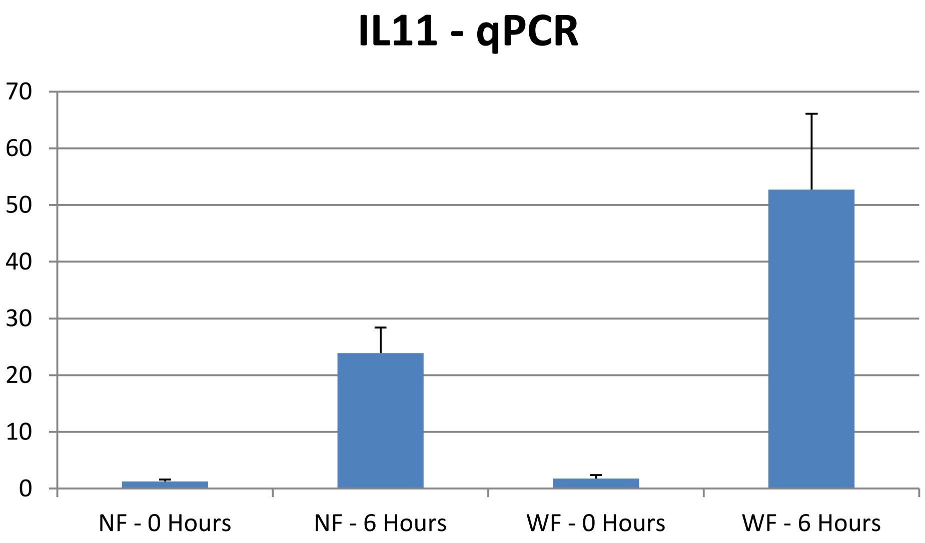
IL11 - qPCR
70
60
50
40
30
20
10
0
NF - 0 Hours
NF - 6 Hours
WF - 0 Hours
WF - 6 Hours

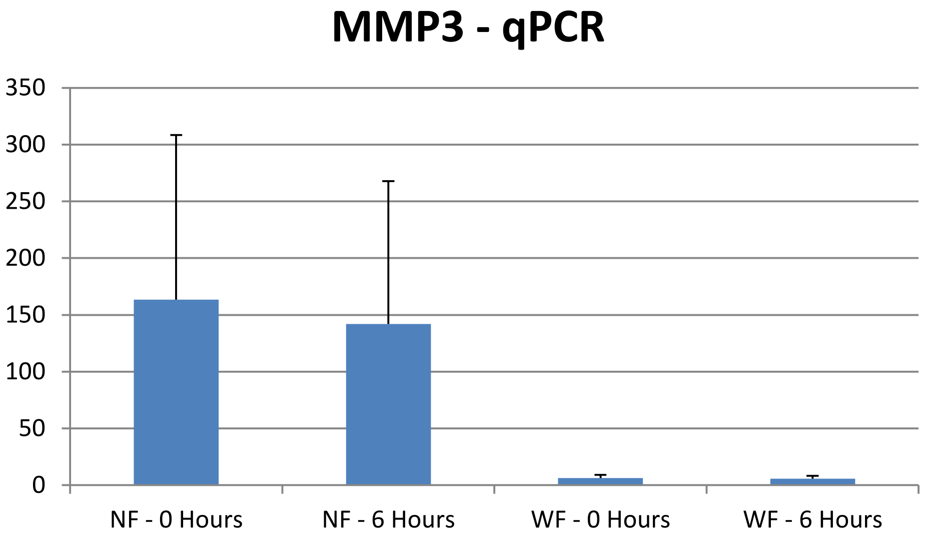
MMP3 - qPCR
350
300
250
200
150
100
50
0
NF - 0 Hours
NF - 6 Hours
WF - 0 Hours
WF - 6 Hours

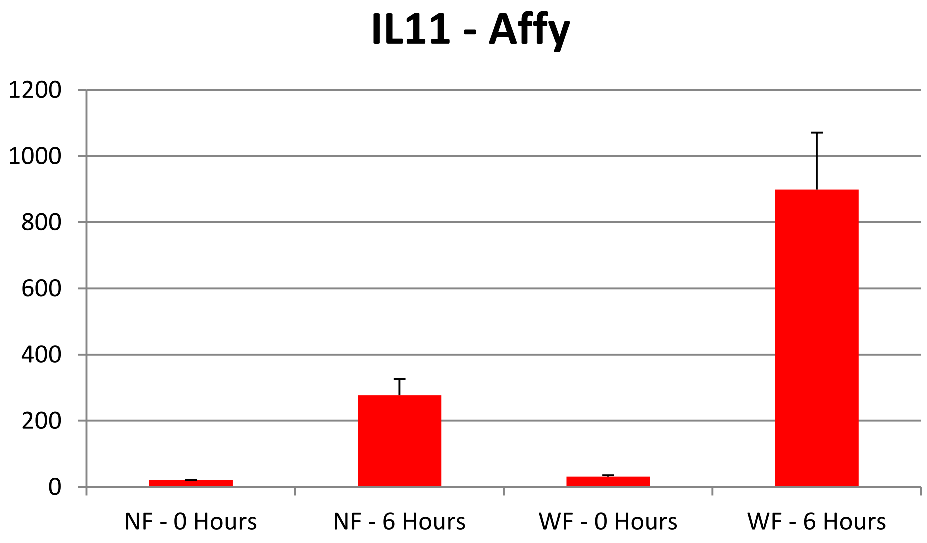
IL11 - Affy
1200
1000
800
600
400
200
0
NF - 0 Hours
NF - 6 Hours
WF - 0 Hours
WF - 6 Hours

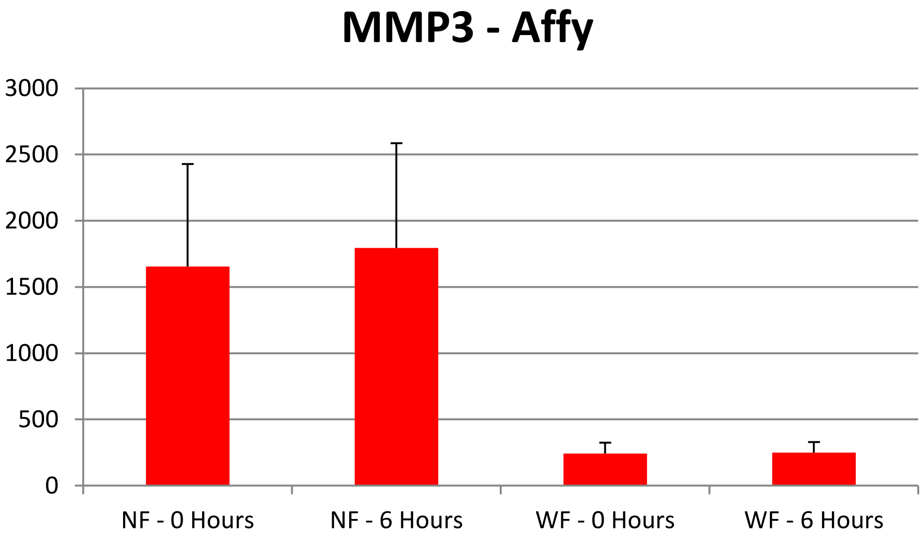
MMP3 - Affy
3000
2500
2000
1500
1000
500
0
NF - 0 Hours
NF - 6 Hours
WF - 0 Hours
WF - 6 Hours
