# Supplementary material for: Identification of a transcriptional signature for the wound healing continuum
Source: Wound Repair Regen. 2014 May 20;22(3):399–405. doi: 10.1111/wrr.12170 (PMC4230470; doi:10.1111/wrr.12170)
Supplement: Supplementary file 2 [file wrr0022-0399-SD2.pdf]

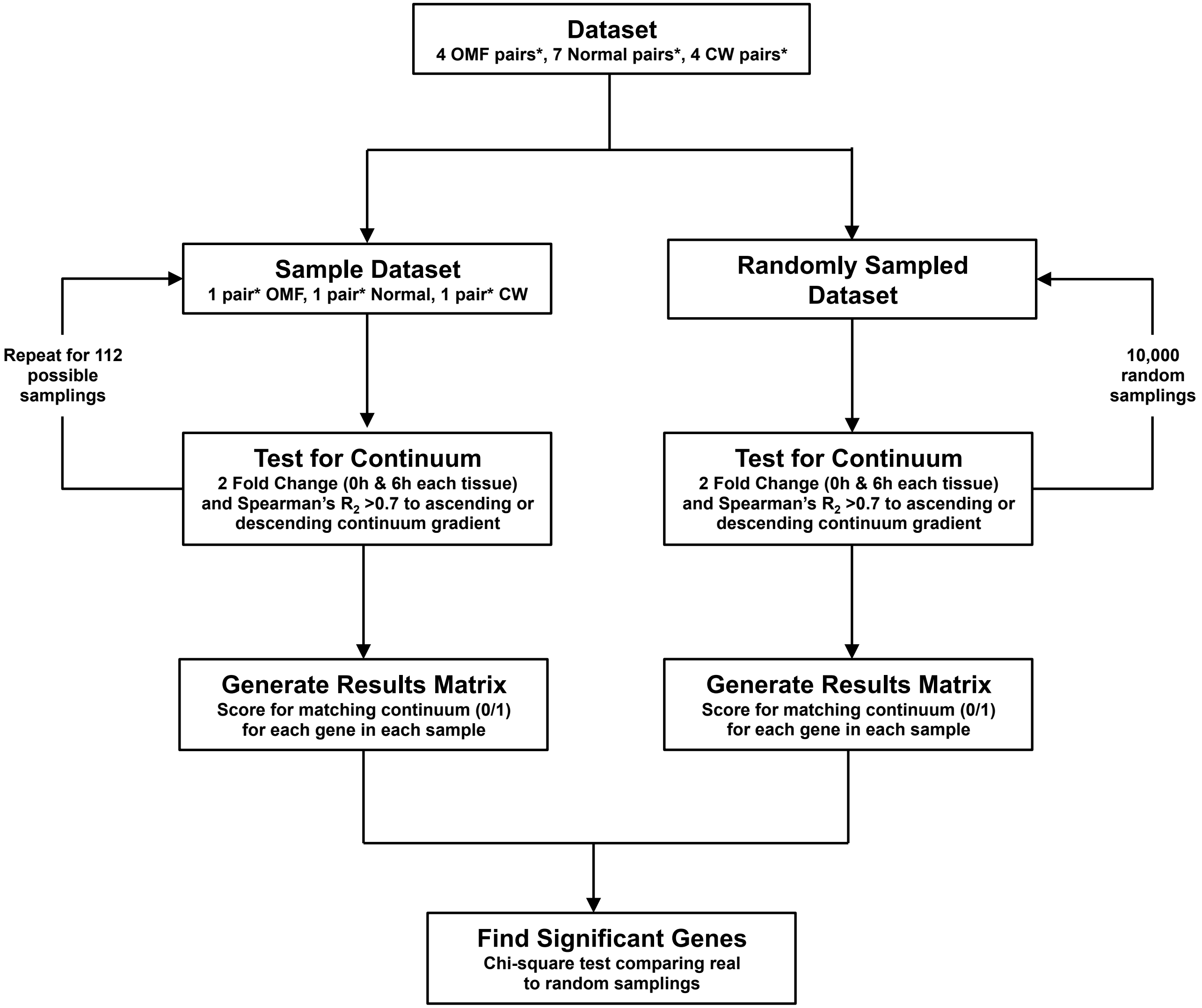

* A pair is a serum starved and a serum stimulated sample from the same patient

Figure 2S – schematic of the continuum analysis methodology
