# Supplementary material for: Identification of a transcriptional signature for the wound healing continuum
Source: Wound Repair Regen. 2014 May 20;22(3):399–405. doi: 10.1111/wrr.12170 (PMC4230470; doi:10.1111/wrr.12170)
Supplement: Supplementary file 3 [file wrr0022-0399-SD3.pdf]

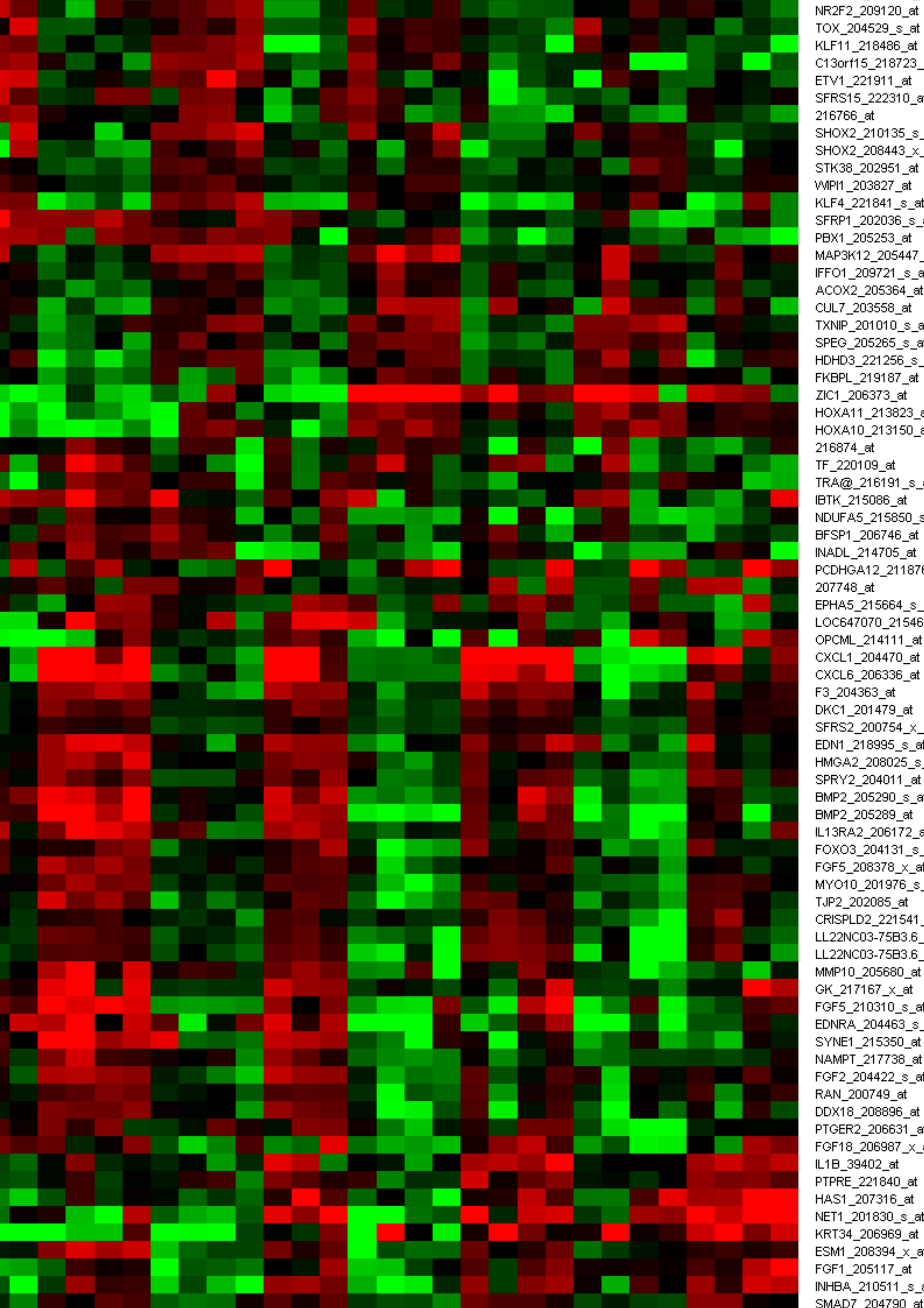
NR2F2_209120_at
TOX_204529_s_at
KLF11_218486_at
C13orf15_218723_
ETV1_221911_at
SFRS15_222310_a
216766_at
SHOX2_210135_s_
SHOX2_208443_x_
STK38_202951_at
WIPI1_203827_at
KLF4_221841_s_a
SFRP1_202036_s_
PBX1_205253_at
MAP3K12_205447_
IFFO1_209721_s_a
ACOX2_205364_at
CUL7_203558_at
TXNIP_201010_s_a
SPEG_205265_s_a
HDHD3_221256_s_
FKBPL_219187_at
ZIC1_206373_at
HOXA11_213823_a
HOXA10_213150_a
216874_at
TF_220109_at
TRA@_216191_s_a
IBTK_215086_at
NDUFA5_215850_s
BFSP1_206746_at
INADL_214705_at
PCDHGA12_21187
207748_at
EPHA5_215664_s_
LOC647070_21546
OPCML_214111_at
CXCL1_204470_at
CXCL6_206336_at
F3_204363_at
DKC1_201479_at
SFRS2_200754_x_
EDN1_218995_s_a
HMGA2_208025_s_
SPRY2_204011_at
BMP2_205290_s_a
BMP2_205289_at
IL13RA2_206172_a
FOXO3_204131_s_
FGF5_208378_x_a
MYO10_201976_s_
TJP2_202085_at
CRISPLD2_221541_
LL22NC03-75B3.6_
LL22NC03-75B3.6_
MMP10_205680_at
GK_217167_x_at
FGF5_210310_s_a
EDNRA_204463_s_
SYNE1_215350_at
NAMPT_217738_at
FGF2_204422_s_a
RAN_200749_at
DDX18_208896_at
PTGER2_206631_a
FGF18_206987_x_
IL1B_39402_at
PTPRE_221840_at
HAS1_207316_at
NET1_201830_s_a
KRT34_206969_at
ESM1_208394_x_a
FGF1_205117_at
INHBA_210511_s_a
SMAD7_204790_at
