# Supplementary material for: Identification of a transcriptional signature for the wound healing continuum
Source: Wound Repair Regen. 2014 May 20;22(3):399–405. doi: 10.1111/wrr.12170 (PMC4230470; doi:10.1111/wrr.12170)
Supplement: Supplementary file 4 [file wrr0022-0399-SD4.pdf]

Table 1S – Patient/tissue details

Patient Sample	Sex	Age biopsy obtained	Site of Skin Biopsy	Site of Oral Mucosal Biopsy	Site of Chronic Wound Biopsy
OMF1/NF1	F	56	Arm	Buccal Mucosa	-
OMF2/NF1	M	77	Arm	Buccal Mucosa	-
OMF3/NF3	M	48	Arm	Buccal Mucosa	-
OMF4/NF4	F	89	Arm	Buccal Mucosa	-
CWF1/NF5	F	71	Thigh	-	Centre of Chronic venous Leg ulcer
CWF2/NF6	F	75	Thigh	-	Centre of Chronic venous Leg ulcer
CWF3/NF7	F	78	Thigh	-	Centre of Chronic venous Leg ulcer
CWF4/NF6	F	68	Thigh	-	Centre of Chronic venous Leg ulcer
