# Supplementary material for: Identification of a transcriptional signature for the wound healing continuum
Source: Wound Repair Regen. 2014 May 20;22(3):399–405. doi: 10.1111/wrr.12170 (PMC4230470; doi:10.1111/wrr.12170)
Supplement: Supplementary file 5 [file wrr0022-0399-SD5.pdf]

Table 2S - Preferential wound healing genes

Affymetrix ID	Gene Name
212614_AT	AT RICH INTERACTIVE DOMAIN 5B (MRF1-LIKE)
218694_AT	ARMADILLO REPEAT CONTAINING, X-LINKED 1
206746_AT	BEADED FILAMENT STRUCTURAL PROTEIN 1, FILENSIN
205289_AT; 205290_S_AT	BONE MORPHOGENETIC PROTEIN 2
218723_S_AT	RESPONSE GENE TO COMPLEMENT 32
201853_S_AT	CELL DIVISION CYCLE 25B
213348_AT; 216894_X_AT	CYCLIN-DEPENDENT KINASE INHIBITOR 1C (P57, KIP2)
221541_AT	CYSTEINE-RICH SECRETORY PROTEIN LCCL DOMAIN CONTAINING 2
204470_AT	CHEMOKINE (C-X-C MOTIF) LIGAND 1 (MELANOMA GROWTH STIMULATING ACTIVITY, ALPHA)
206336_AT	CHEMOKINE (C-X-C MOTIF) LIGAND 6 (GRANULOCYTE CHEMOTACTIC PROTEIN 2)
207748_AT	ASPARTYL-TRNA SYNTHETASE
208896_AT	DEAD (ASP-GLU-ALA-ASP) BOX POLYPEPTIDE 18
201479_AT	DYSKERATOSIS CONGENITA 1, DYSKERIN
218995_S_AT	ENDOTHELIN 1
204463_S_AT	ENDOTHELIN RECEPTOR TYPE A
215664_S_AT	EPH RECEPTOR A5
208394_X_AT	ENDOTHELIAL CELL-SPECIFIC MOLECULE 1
221911_AT	ETS VARIANT GENE 1
204363_AT	COAGULATION FACTOR III (THROMBOPLASTIN, TISSUE FACTOR)
217047_S_AT	FAMILY WITH SEQUENCE SIMILARITY 13, MEMBER A1
206987_X_AT; 211029_X_AT	FIBROBLAST GROWTH FACTOR 18
208378_X_AT; 210310_S_AT	FIBROBLAST GROWTH FACTOR 5
204131_S_AT	FORKHEAD BOX O3A
215966_X_AT; 217167_X_AT	GLYCEROL KINASE
204396_S_AT	G PROTEIN-COUPLED RECEPTOR KINASE 5
208025_S_AT	HIGH MOBILITY GROUP AT-HOOK 2
215086_AT	INHIBITOR OF BRUTON AGAMMAGLOBULINEMIA TYROSINE KINASE
206172_AT	INTERLEUKIN 13 RECEPTOR, ALPHA 2
214705_AT	INAD-LIKE (DROSOPHILA)
218486_AT	KRUPPEL-LIKE FACTOR 11
221841_S_AT	KRUPPEL-LIKE FACTOR 4 (GUT)

221901_AT; 52837_AT	KIAA1644 PROTEIN
212276_AT	LIPIN 1
206163_AT	MAB-21-LIKE 1 (C. ELEGANS)
210302_S_AT	MAB-21-LIKE 2 (C. ELEGANS)
206363_AT; 209348_S_AT	V-MAF MUSCULOAPONEUROTIC FIBROSARCOMA ONCOGENE HOMOLOG (AVIAN)
205680_AT	MATRIX METALLOPEPTIDASE 10 (STROMELYSIN 2)
220319_S_AT	MYOSIN REGULATORY LIGHT CHAIN INTERACTING PROTEIN
201976_S_AT	MYOSIN X
221207_S_AT	NEUROBEACHIN
215850_S_AT	NADH DEHYDROGENASE (UBIQUINONE) 1 ALPHA SUBCOMPLEX, 5, 13KDA
209120_AT	NUCLEAR RECEPTOR SUBFAMILY 2, GROUP F, MEMBER 2
213568_AT	ODD-SKIPPED RELATED 2 (DROSOPHILA)
217738_AT	PRE-B-CELL COLONY ENHANCING FACTOR 1
205253_AT	PRE-B-CELL LEUKEMIA TRANSCRIPTION FACTOR 1
203131_AT	PLATELET-DERIVED GROWTH FACTOR RECEPTOR, ALPHA POLYPEPTIDE
219132_AT	PELLINO HOMOLOG 2 (DROSOPHILA)
216766_AT	PROTEIN KINASE C, EPSILON
209815_AT	PATCHED HOMOLOG (DROSOPHILA)
206631_AT	PROSTAGLANDIN E RECEPTOR 2 (SUBTYPE EP2), 53KDA
200749_AT	RAN, MEMBER RAS ONCOGENE FAMILY
208070_S_AT	REV3-LIKE, CATALYTIC SUBUNIT OF DNA POLYMERASE ZETA (YEAST)
209568_S_AT	RAL GUANINE NUCLEOTIDE DISSOCIATION STIMULATOR-LIKE 1
202036_S_AT	SECRETED FRIZZLED-RELATED PROTEIN 1
222310_AT	SPLICING FACTOR, ARGININE/SERINE-RICH 15
200754_X_AT	SPLICING FACTOR, ARGININE/SERINE-RICH 2
208443_X_AT; 210135_S_AT	SHORT STATURE HOMEOBOX 2
204011_AT	SPROUTY HOMOLOG 2 (DROSOPHILA)
215350_AT	SPECTRIN REPEAT CONTAINING, NUCLEAR ENVELOPE 1
204731_AT	TRANSFORMING GROWTH FACTOR, BETA RECEPTOR III (BETAGLYCAN, 300KDA)
202085_AT	TIGHT JUNCTION PROTEIN 2 (ZONA OCCLUDENS 2)
204529_S_AT	THYMUS HIGH MOBILITY GROUP BOX PROTEIN TOX
213943_AT	TWIST HOMOLOG 1 (ACROCEPHALOSYNDACTYLY 3; SAETHRE-CHOTZEN SYNDROME) (DROSOPHILA)
203827_AT	WD REPEAT DOMAIN, PHOSPHOINOSITIDE INTERACTING 1
201367_S_AT; 201368_AT; 201369_S_AT	ZINC FINGER PROTEIN 36, C3H TYPE-LIKE 2

218149_S_AT; 221123_X_AT	HYPOTHETICAL PROTEIN DKFZP434K1210
200749_AT; 213817_AT	NA

GENE_SYMBOL
ARID5B
ARMCX1
BFSP1
BMP2
C13ORF15
CDC25B
CDKN1C
CRISPLD2
CXCL1
CXCL6
DARS
DDX18
DKC1
EDN1
EDNRA
EPHA5
ESM1
ETV1
F3
FAM13A1
FGF18
FGF5
FOXO3
GK
GRK5
HMGA2
IBTK
IL13RA2
INADL
KLF11
KLF4

LL22NC03-75B3.6
LPIN1
MAB21L1
MAB21L2
MAF
MMP10
MYLIP
MYO10
NBEA
NDUFA5
NR2F2
OSR2
PBEF1
PBX1
PDGFRA
PELI2
PRKCE
PTCH1
PTGER2
RAN
REV3L
RGL1
SFRP1
SFRS15
SFRS2
SHOX2
SPRY2
SYNE1
TGFBR3
TJP2
TOX
TWIST1
WIPI1
ZFP36L2

ZNF395

NA
