# Supplementary material for: Identification of a transcriptional signature for the wound healing continuum
Source: Wound Repair Regen. 2014 May 20;22(3):399–405. doi: 10.1111/wrr.12170 (PMC4230470; doi:10.1111/wrr.12170)
Supplement: Supplementary file 6 [file wrr0022-0399-SD6.pdf]

Table 3S - Dysfunctional wound healing genes

Affymetrix ID	Gene Name	GENE_SYMBOL
205364_AT	ACYL-COENZYME A OXIDASE 2, BRANCHED CHAIN	ACOX2
209765_AT	ADAM METALLOPEPTIDASE DOMAIN 19 (MELTRIN BETA)	ADAM19
214255_AT	ATPASE, CLASS V, TYPE 10C	ATP10A
203612_AT	BYSTIN-LIKE	BYSL
212419_AT; 212423_AT	CHROMOSOME 10 OPEN READING FRAME 56	C10ORF56
203558_AT	CULLIN 7	CUL7
201041_S_AT	DUAL SPECIFICITY PHOSPHATASE 1	DUSP1
205117_AT	FIBROBLAST GROWTH FACTOR 1 (ACIDIC)	FGF1
219187_AT	FK506 BINDING PROTEIN LIKE	FKBPL
207876_S_AT	FILAMIN C, GAMMA (ACTIN BINDING PROTEIN 280)	FLNC
207574_S_AT; 209304_X_AT	GROWTH ARREST AND DNA-DAMAGE-INDUCIBLE, BETA	GADD45B
212432_AT	GRPE-LIKE 1, MITOCHONDRIAL (E. COLI)	GRPEL1
207316_AT	HYALURONAN SYNTHASE 1	HAS1
221256_S_AT	HALOACID DEHALOGENASE-LIKE HYDROLASE DOMAIN CONTAINING 3	HDHD3
209721_S_AT	HOM-TES-103 TUMOR ANTIGEN-LIKE	HOM-TES-103
213150_AT	HOMEOBOX A10	HOXA10
213823_AT	HOMEOBOX A11	HOXA11
201631_S_AT	IMMEDIATE EARLY RESPONSE 3	IER3
205067_AT; 39402_AT	INTERLEUKIN 1, BETA	IL1B
210511_S_AT	INHIBIN, BETA A (ACTIVIN A, ACTIVIN AB ALPHA POLYPEPTIDE)	INHBA
206969_AT	KERATIN, HAIR, ACIDIC, 4	KRT34
205447_S_AT	MITOGEN-ACTIVATED PROTEIN KINASE KINASE KINASE 12	MAP3K12
212472_AT; 212473_S_AT	MICROTUBULE ASSOCIATED MONOXYGENASE, CALPONIN AND LIM DOMAIN CONTAINING 2	MICAL2
220319_S_AT	MYOSIN REGULATORY LIGHT CHAIN INTERACTING PROTEIN	MYLIP
201830_S_AT	NEUROEPITHELIAL CELL TRANSFORMING GENE 1	NET1
214111_AT	OPIOID BINDING PROTEIN/CELL ADHESION MOLECULE-LIKE	OPCML
203879_AT	PHOSPHOINOSITIDE-3-KINASE, CATALYTIC, DELTA POLYPEPTIDE	PIK3CD
204195_S_AT	PBX/KNOTTED 1 HOMEOBOX 1	PKNOX1
208447_S_AT; 209440_AT	PHOSPHORIBOSYL PYROPHOSPHATE SYNTHETASE 1	PRPS2
202627_S_AT; 202628_S_AT	SERPIN PEPTIDASE INHIBITOR, CLADE E (NEXIN, PLASMINOGEN ACTIVATOR INHIBITOR TYPE 1), MEMBER 1	SERPINE1
204790_AT	SMAD, MOTHERS AGAINST DPP HOMOLOG 7 (DROSOPHILA)	SMAD7

205265_S_AT	AORTIC PREFERENTIALLY EXPRESSED GENE 1	SPEG
219257_S_AT	SPHINGOSINE KINASE 1	SPHK1
216191_S_AT	T CELL RECEPTOR ALPHA LOCUS	TRA@
216191_S_AT	T CELL RECEPTOR DELTA LOCUS	TRD@
201010_S_AT	THIOREDOXIN INTERACTING PROTEIN	TXNIP
209825_S_AT	URIDINE-CYTIDINE KINASE 2	UCK2
206373_AT	ZIC FAMILY MEMBER 1 (ODD-PAIRED HOMOLOG, DROSOPHILA)	ZIC1
