# Supplementary material for: Identification of a transcriptional signature for the wound healing continuum
Source: Wound Repair Regen. 2014 May 20;22(3):399–405. doi: 10.1111/wrr.12170 (PMC4230470; doi:10.1111/wrr.12170)
Supplement: Supplementary file 7 [file wrr0022-0399-SD7.pdf]

Table 4S - Functional classification of wound healing continuum genes

Gene Ontology ID	Functional Classification	Count	%	PValue	Probe Ids
GO:0042127	regulation of cell proliferation	27	25.471698	4.91E-12	204470_AT, 39402_AT, 221841_S_AT, 201853_S_AT, 204463_S_AT, 210302_S_AT, 203131_AT, 205265_S_AT, 218995_S_AT, 219187_AT, 205117_AT, 209815_AT, 204422_S_AT, 206163_AT, 202628_S_AT, 201010_S_AT, 217738_AT, 206631_AT, 206987_X_AT, 205253_AT, 213348_AT, 218486_AT, 219257_S_AT, 213568_AT, 202627_S_AT, 204363_AT, 205289_AT, 205290_S_AT, 204731_AT
GO:0048856	anatomical structure development	45	42.45283	1.04E-10	221841_S_AT, 209120_AT, 204463_S_AT, 214111_AT, 210302_S_AT, 204195_S_AT, 209440_AT, 205265_S_AT, 208447_S_AT, 221541_AT, 203558_AT, 213150_AT, 209348_S_AT, 220319_S_AT, 206163_AT, 208443_X_AT, 213943_AT, 201010_S_AT, 213823_AT, 208378_X_AT, 213348_AT, 204790_AT, 206969_AT, 202036_S_AT, 209765_AT, 210511_S_AT, 206363_AT, 205289_AT, 204011_AT, 204470_AT, 204131_S_AT, 39402_AT, 215350_AT, 203131_AT, 218995_S_AT, 219187_AT, 205117_AT, 209815_AT, 204422_S_AT, 202628_S_AT, 206987_X_AT, 205253_AT, 210135_S_AT, 212614_AT, 206373_AT, 201631_S_AT, 213568_AT, 202627_S_AT, 210310_S_AT, 205290_S_AT, 204731_AT

GO:0048731	system development	43	40.566038	1.23E-10	221841_S_AT, 209120_AT, 204463_S_AT, 214111_AT, 210302_S_AT, 204195_S_AT, 209440_AT, 205265_S_AT, 208447_S_AT, 221541_AT, 203558_AT, 213150_AT, 209348_S_AT, 220319_S_AT, 206163_AT, 208443_X_AT, 213943_AT, 201010_S_AT, 213823_AT, 208378_X_AT, 213348_AT, 204790_AT, 206969_AT, 202036_S_AT, 209765_AT, 210511_S_AT, 206363_AT, 205289_AT, 204011_AT, 204470_AT, 204131_S_AT, 39402_AT, 203131_AT, 218995_S_AT, 205117_AT, 219187_AT, 209815_AT, 204422_S_AT, 202628_S_AT, 206987_X_AT, 205253_AT, 210135_S_AT, 212614_AT, 206373_AT, 213568_AT, 202627_S_AT, 210310_S_AT, 205290_S_AT, 204731_AT
GO:0048513	organ development	36	33.962264	5.13E-10	204011_AT, 39402_AT, 204131_S_AT, 221841_S_AT, 209120_AT, 210302_S_AT, 204463_S_AT, 204195_S_AT, 203131_AT, 205265_S_AT, 218995_S_AT, 219187_AT, 205117_AT, 221541_AT, 203558_AT, 209815_AT, 209348_S_AT, 204422_S_AT, 206163_AT, 208443_X_AT, 213943_AT, 202628_S_AT, 201010_S_AT, 213823_AT, 206987_X_AT, 205253_AT, 210135_S_AT, 212614_AT, 204790_AT, 206969_AT, 206373_AT, 202036_S_AT, 213568_AT, 202627_S_AT, 209765_AT, 210511_S_AT, 206363_AT, 204731_AT, 205290_S_AT, 205289_AT

GO:0032502	developmental process	49	46.226415	8.92E-10	208025_S_AT, 221841_S_AT, 209120_AT, 201853_S_AT, 210302_S_AT, 204463_S_AT, 214111_AT, 209440_AT, 204195_S_AT, 209304_X_AT, 208447_S_AT, 205265_S_AT, 221541_AT, 203558_AT, 213150_AT, 209348_S_AT, 220319_S_AT, 206163_AT, 208443_X_AT, 213943_AT, 201010_S_AT, 213823_AT, 208378_X_AT, 213348_AT, 204790_AT, 206969_AT, 202036_S_AT, 209765_AT, 210511_S_AT, 206363_AT, 205289_AT, 204011_AT, 207574_S_AT, 204470_AT, 204131_S_AT, 39402_AT, 215350_AT, 203131_AT, 218995_S_AT, 219187_AT, 205117_AT, 209815_AT, 204422_S_AT, 200749_AT, 202628_S_AT, 206987_X_AT, 205253_AT, 210135_S_AT, 212614_AT, 206373_AT, 201631_S_AT, 213568_AT, 202627_S_AT, 210310_S_AT, 204731_AT, 205290_S_AT

GO:0007275	multicellular organismal development	45	42.45283	6.49E-09	208025_S_AT, 221841_S_AT, 209120_AT, 204463_S_AT, 214111_AT, 210302_S_AT, 204195_S_AT, 209440_AT, 208447_S_AT, 205265_S_AT, 209304_X_AT, 221541_AT, 203558_AT, 213150_AT, 209348_S_AT, 220319_S_AT, 206163_AT, 208443_X_AT, 213943_AT, 201010_S_AT, 213823_AT, 208378_X_AT, 213348_AT, 204790_AT, 206969_AT, 202036_S_AT, 209765_AT, 210511_S_AT, 206363_AT, 205289_AT, 204011_AT, 207574_S_AT, 204470_AT, 204131_S_AT, 39402_AT, 203131_AT, 218995_S_AT, 219187_AT, 205117_AT, 209815_AT, 204422_S_AT, 202628_S_AT, 206987_X_AT, 205253_AT, 210135_S_AT, 212614_AT, 206373_AT, 213568_AT, 202627_S_AT, 210310_S_AT, 205290_S_AT, 204731_AT
GO:0009653	anatomical structure morphogenesis	28	26.415094	9.17E-09	204011_AT, 39402_AT, 221841_S_AT, 209120_AT, 204463_S_AT, 210302_S_AT, 204195_S_AT, 203131_AT, 218995_S_AT, 205117_AT, 203558_AT, 213150_AT, 209815_AT, 204422_S_AT, 206163_AT, 213943_AT, 202628_S_AT, 213823_AT, 206987_X_AT, 205253_AT, 212614_AT, 204790_AT, 206373_AT, 202036_S_AT, 201631_S_AT, 213568_AT, 202627_S_AT, 205289_AT, 205290_S_AT, 204731_AT
GO:0035295	tube development	13	12.264151	2.36E-08	204011_AT, 213823_AT, 204463_S_AT, 206987_X_AT, 205253_AT, 203131_AT, 218995_S_AT, 205117_AT, 221541_AT, 209815_AT, 204422_S_AT, 213943_AT, 205290_S_AT, 205289_AT

GO:0050793	regulation of developmental process	20	18.867925	7.65E-08	213823_AT, 204131_S_AT, 39402_AT, 221841_S_AT, 212276_AT, 220109_AT, 205253_AT, 218995_S_AT, 204790_AT, 202036_S_AT, 219257_S_AT, 209348_S_AT, 214255_AT, 204422_S_AT, 204363_AT, 202627_S_AT, 210511_S_AT, 206363_AT, 213943_AT, 202628_S_AT, 204731_AT, 205290_S_AT, 205289_AT
GO:0030154	cell differentiation	30	28.301887	4.51E-07	204011_AT, 207574_S_AT, 204131_S_AT, 215350_AT, 221841_S_AT, 209120_AT, 201853_S_AT, 204463_S_AT, 214111_AT, 204195_S_AT, 203131_AT, 209304_X_AT, 205265_S_AT, 218995_S_AT, 205117_AT, 203558_AT, 209348_S_AT, 204422_S_AT, 200749_AT, 213943_AT, 201010_S_AT, 213823_AT, 206987_X_AT, 205253_AT, 208378_X_AT, 213348_AT, 206373_AT, 202036_S_AT, 210310_S_AT, 210511_S_AT, 206363_AT, 204731_AT, 205290_S_AT, 205289_AT
GO:0048522	positive regulation of cellular process	32	30.188679	5.12E-07	39402_AT, 204131_S_AT, 221841_S_AT, 209120_AT, 201853_S_AT, 210302_S_AT, 204463_S_AT, 220109_AT, 204195_S_AT, 203131_AT, 218995_S_AT, 201041_S_AT, 219187_AT, 205117_AT, 201830_S_AT, 209348_S_AT, 204422_S_AT, 206163_AT, 200749_AT, 201010_S_AT, 213823_AT, 217738_AT, 206987_X_AT, 205253_AT, 208378_X_AT, 213348_AT, 204790_AT, 219257_S_AT, 213568_AT, 204363_AT, 210310_S_AT, 210511_S_AT, 206363_AT, 205289_AT, 205290_S_AT

GO:0008284	positive regulation of cell proliferation	15	14.150943	5.75E-07	39402_AT, 217738_AT, 201853_S_AT, 210302_S_AT, 206987_X_AT, 205253_AT, 203131_AT, 218995_S_AT, 219187_AT, 205117_AT, 219257_S_AT, 213568_AT, 204363_AT, 204422_S_AT, 206163_AT
GO:0048869	cellular developmental process	30	28.301887	1.07E-06	204011_AT, 207574_S_AT, 204131_S_AT, 215350_AT, 221841_S_AT, 209120_AT, 201853_S_AT, 204463_S_AT, 214111_AT, 204195_S_AT, 203131_AT, 209304_X_AT, 205265_S_AT, 218995_S_AT, 205117_AT, 203558_AT, 209348_S_AT, 204422_S_AT, 200749_AT, 213943_AT, 201010_S_AT, 213823_AT, 206987_X_AT, 205253_AT, 208378_X_AT, 213348_AT, 206373_AT, 202036_S_AT, 210310_S_AT, 210511_S_AT, 206363_AT, 204731_AT, 205290_S_AT, 205289_AT
GO:0048518	positive regulation of biological process	32	30.188679	4.10E-06	39402_AT, 204131_S_AT, 221841_S_AT, 209120_AT, 201853_S_AT, 210302_S_AT, 204463_S_AT, 220109_AT, 204195_S_AT, 203131_AT, 218995_S_AT, 201041_S_AT, 219187_AT, 205117_AT, 201830_S_AT, 209348_S_AT, 204422_S_AT, 206163_AT, 200749_AT, 201010_S_AT, 213823_AT, 217738_AT, 206987_X_AT, 205253_AT, 208378_X_AT, 213348_AT, 204790_AT, 219257_S_AT, 213568_AT, 204363_AT, 210310_S_AT, 210511_S_AT, 206363_AT, 205289_AT, 205290_S_AT

GO:0009887	organ morphogenesis	16	15.09434	4.39E-06	204011_AT, 213823_AT, 221841_S_AT, 206987_X_AT, 205253_AT, 203131_AT, 218995_S_AT, 204790_AT, 212614_AT, 206373_AT, 209815_AT, 213568_AT, 204422_S_AT, 213943_AT, 204731_AT, 205290_S_AT, 205289_AT
GO:0009790	embryonic development	16	15.09434	4.79E-06	204011_AT, 213823_AT, 221841_S_AT, 210302_S_AT, 204463_S_AT, 205253_AT, 203131_AT, 218995_S_AT, 206373_AT, 202036_S_AT, 213150_AT, 209815_AT, 213568_AT, 213943_AT, 204731_AT, 205290_S_AT, 205289_AT
GO:0001568	blood vessel development	11	10.377358	5.20E-06	205117_AT, 204790_AT, 203558_AT, 39402_AT, 209120_AT, 204422_S_AT, 204463_S_AT, 206987_X_AT, 204195_S_AT, 204731_AT, 218995_S_AT
GO:0001944	vasculature development	11	10.377358	6.44E-06	205117_AT, 204790_AT, 203558_AT, 39402_AT, 209120_AT, 204422_S_AT, 204463_S_AT, 206987_X_AT, 204195_S_AT, 204731_AT, 218995_S_AT
GO:0001501	skeletal system development	12	11.320755	8.59E-06	213823_AT, 206987_X_AT, 205253_AT, 203131_AT, 218995_S_AT, 210135_S_AT, 212614_AT, 213150_AT, 213568_AT, 210511_S_AT, 208443_X_AT, 213943_AT, 205290_S_AT, 205289_AT
GO:0048514	blood vessel morphogenesis	10	9.4339623	1.11E-05	205117_AT, 204790_AT, 203558_AT, 39402_AT, 209120_AT, 204422_S_AT, 204463_S_AT, 206987_X_AT, 204195_S_AT, 218995_S_AT

GO:0042325	regulation of phosphorylation	14	13.207547	1.23E-05	204011_AT, 218723_S_AT, 207574_S_AT, 39402_AT, 201853_S_AT, 204463_S_AT, 209304_X_AT, 218995_S_AT, 213348_AT, 204790_AT, 219257_S_AT, 204422_S_AT, 210511_S_AT, 215086_AT, 205290_S_AT, 205289_AT
GO:0050794	regulation of cellular process	67	63.207547	1.31E-05	208025_S_AT, 221841_S_AT, 209120_AT, 201853_S_AT, 210302_S_AT, 204463_S_AT, 204195_S_AT, 209304_X_AT, 205265_S_AT, 201041_S_AT, 203558_AT, 201830_S_AT, 213150_AT, 209348_S_AT, 214255_AT, 206163_AT, 214705_AT, 217047_S_AT, 208443_X_AT, 213943_AT, 218149_S_AT, 201010_S_AT, 205447_S_AT, 213823_AT, 209568_S_AT, 217738_AT, 221911_AT, 212276_AT, 206336_AT, 201369_S_AT, 213348_AT, 208378_X_AT, 204790_AT, 202036_S_AT, 218486_AT, 204363_AT, 210511_S_AT, 206363_AT, 205289_AT, 204011_AT, 218723_S_AT, 207574_S_AT, 204470_AT, 204131_S_AT, 39402_AT, 220109_AT, 203131_AT, 208394_X_AT, 218995_S_AT, 219187_AT, 205117_AT, 209815_AT, 204422_S_AT, 200749_AT, 202628_S_AT, 203879_AT, 206631_AT, 206987_X_AT, 205253_AT, 210135_S_AT, 212614_AT, 206373_AT, 201976_S_AT, 219257_S_AT, 201631_S_AT, 204396_S_AT, 202951_AT, 213568_AT, 202627_S_AT, 210310_S_AT, 215086_AT, 204731_AT, 205290_S_AT

GO:0007167	enzyme linked receptor protein signaling pathway	12	11.320755	1.65E-05	221840_AT, 206987_X_AT, 203131_AT, 205117_AT, 208378_X_AT, 215664_S_AT, 204790_AT, 212614_AT, 204422_S_AT, 210310_S_AT, 201010_S_AT, 205290_S_AT, 205289_AT, 204731_AT
GO:0051174	regulation of phosphorus metabolic process	14	13.207547	1.88E-05	204011_AT, 218723_S_AT, 207574_S_AT, 39402_AT, 201853_S_AT, 204463_S_AT, 209304_X_AT, 218995_S_AT, 213348_AT, 204790_AT, 219257_S_AT, 204422_S_AT, 210511_S_AT, 215086_AT, 205290_S_AT, 205289_AT
GO:0019220	regulation of phosphate metabolic process	14	13.207547	1.88E-05	204011_AT, 218723_S_AT, 207574_S_AT, 39402_AT, 201853_S_AT, 204463_S_AT, 209304_X_AT, 218995_S_AT, 213348_AT, 204790_AT, 219257_S_AT, 204422_S_AT, 210511_S_AT, 215086_AT, 205290_S_AT, 205289_AT

GO:0032501	multicellular organismal process	49	46.226415	2.00E-05	208025_S_AT, 221841_S_AT, 209120_AT, 201853_S_AT, 210302_S_AT, 204463_S_AT, 214111_AT, 209440_AT, 204195_S_AT, 209304_X_AT, 208447_S_AT, 205265_S_AT, 221541_AT, 203558_AT, 213150_AT, 209348_S_AT, 220319_S_AT, 206163_AT, 208443_X_AT, 213943_AT, 201010_S_AT, 205680_AT, 213823_AT, 208378_X_AT, 213348_AT, 204790_AT, 206969_AT, 202036_S_AT, 204363_AT, 209765_AT, 210511_S_AT, 206363_AT, 205289_AT, 204011_AT, 207574_S_AT, 204470_AT, 204131_S_AT, 39402_AT, 203131_AT, 218995_S_AT, 219187_AT, 205117_AT, 209815_AT, 204422_S_AT, 200749_AT, 202628_S_AT, 206987_X_AT, 205253_AT, 210135_S_AT, 212614_AT, 206373_AT, 213568_AT, 202627_S_AT, 210310_S_AT, 204731_AT, 205290_S_AT
GO:0048646	anatomical structure formation involved in morphogenes	12	11.320755	2.15E-05	205117_AT, 39402_AT, 202036_S_AT, 221841_S_AT, 209815_AT, 204422_S_AT, 204463_S_AT, 206987_X_AT, 204195_S_AT, 213943_AT, 204731_AT, 218995_S_AT
GO:0035239	tube morphogenesis	8	7.5471698	2.16E-05	204011_AT, 213823_AT, 209815_AT, 204463_S_AT, 205253_AT, 213943_AT, 218995_S_AT, 205289_AT, 205290_S_AT
GO:0048598	embryonic morphogenesis	11	10.377358	3.68E-05	204011_AT, 206373_AT, 213823_AT, 221841_S_AT, 213150_AT, 209815_AT, 213568_AT, 210302_S_AT, 205253_AT, 213943_AT, 218995_S_AT

					208025_S_AT, 221841_S_AT, 209120_AT, 201853_S_AT, 210302_S_AT, 204463_S_AT, 204195_S_AT, 209304_X_AT, 205265_S_AT, 201041_S_AT, 203558_AT, 201830_S_AT, 213150_AT, 209348_S_AT, 214255_AT, 206163_AT, 214705_AT, 217047_S_AT, 208443_X_AT, 213943_AT, 218149_S_AT, 201010_S_AT, 205447_S_AT, 213823_AT, 209568_S_AT, 217738_AT, 221911_AT, 212276_AT, 206336_AT, 201369_S_AT, 213348_AT, 208378_X_AT, 204790_AT, 202036_S_AT, 218486_AT, 204363_AT, 210511_S_AT, 206363_AT, 205289_AT, 204011_AT, 218723_S_AT, 207574_S_AT, 204470_AT, 204131_S_AT, 39402_AT, 220109_AT, 203131_AT, 208394_X_AT, 218995_S_AT, 219187_AT, 205117_AT, 209815_AT, 204422_S_AT, 200749_AT, 202628_S_AT, 203879_AT, 206631_AT, 206987_X_AT, 205253_AT, 210135_S_AT, 212614_AT, 206373_AT, 201976_S_AT, 219257_S_AT, 201631_S_AT, 204396_S_AT, 202951_AT, 213568_AT, 202627_S_AT, 210310_S_AT, 215086_AT,
GO:0050789	regulation of biological process	67	63.207547	6.89E-05	204731_AT, 205290_S_AT
GO:0048754	branching morphogenesis of a tube	6	5.6603774	7.09E-05	204011_AT, 213823_AT, 204463_S_AT, 205253_AT, 218995_S_AT, 205289_AT, 205290_S_AT
GO:0045595	regulation of cell differentiation	13	12.264151	9.99E-05	204131_S_AT, 213823_AT, 221841_S_AT, 212276_AT, 205253_AT, 218995_S_AT, 204790_AT, 209348_S_AT, 204422_S_AT, 210511_S_AT, 213943_AT, 206363_AT, 204731_AT, 205290_S_AT, 205289_AT

GO:0022603	regulation of anatomical structure morphogenesis	9	8.490566	1.05E-04	204790_AT, 39402_AT, 219257_S_AT, 214255_AT, 204422_S_AT, 204363_AT, 202627_S_AT, 202628_S_AT, 204731_AT, 218995_S_AT
GO:0050678	regulation of epithelial cell proliferation	6	5.6603774	1.08E-04	205117_AT, 213348_AT, 209815_AT, 204422_S_AT, 204463_S_AT, 204731_AT
GO:0009888	tissue development	15	14.150943	1.22E-04	213823_AT, 221841_S_AT, 209120_AT, 204463_S_AT, 206987_X_AT, 205253_AT, 218995_S_AT, 204790_AT, 206969_AT, 209815_AT, 202627_S_AT, 213943_AT, 202628_S_AT, 204731_AT, 201010_S_AT, 205290_S_AT, 205289_AT
GO:0001763	morphogenesis of a branching structure	6	5.6603774	1.32E-04	204011_AT, 213823_AT, 204463_S_AT, 205253_AT, 218995_S_AT, 205289_AT, 205290_S_AT
GO:0051239	regulation of multicellular organismal process	18	16.981132	1.34E-04	213823_AT, 204131_S_AT, 39402_AT, 220109_AT, 205253_AT, 218995_S_AT, 204790_AT, 202036_S_AT, 209815_AT, 219257_S_AT, 209348_S_AT, 204422_S_AT, 204363_AT, 202627_S_AT, 210511_S_AT, 206363_AT, 213943_AT, 202628_S_AT, 204731_AT, 205290_S_AT, 205289_AT
GO:0007423	sensory organ development	9	8.490566	1.43E-04	204011_AT, 206373_AT, 221841_S_AT, 209348_S_AT, 210302_S_AT, 206163_AT, 204195_S_AT, 206363_AT, 203131_AT, 218995_S_AT
GO:0048729	tissue morphogenesis	8	7.5471698	1.96E-04	204790_AT, 213823_AT, 221841_S_AT, 209815_AT, 205253_AT, 213943_AT, 204731_AT, 205289_AT, 205290_S_AT

GO:0006793	phosphorus metabolic process	18	16.981132	2.10E-04	205447_S_AT, 207574_S_AT, 203879_AT, 217167_X_AT, 39402_AT, 221840_AT, 201853_S_AT, 215850_S_AT, 203131_AT, 205265_S_AT, 209304_X_AT, 201041_S_AT, 215664_S_AT, 204790_AT, 202951_AT, 204396_S_AT, 204422_S_AT, 204731_AT, 205290_S_AT, 205289_AT
GO:0006796	phosphate metabolic process	18	16.981132	2.10E-04	205447_S_AT, 207574_S_AT, 203879_AT, 217167_X_AT, 39402_AT, 221840_AT, 201853_S_AT, 215850_S_AT, 203131_AT, 205265_S_AT, 209304_X_AT, 201041_S_AT, 215664_S_AT, 204790_AT, 202951_AT, 204396_S_AT, 204422_S_AT, 204731_AT, 205290_S_AT, 205289_AT

GO:0065007	biological regulation	68	64.150943	2.20E-04	208025_S_AT, 221841_S_AT, 209120_AT, 201853_S_AT, 210302_S_AT, 204463_S_AT, 201479_AT, 204195_S_AT, 209304_X_AT, 205265_S_AT, 201041_S_AT, 203558_AT, 201830_S_AT, 213150_AT, 209348_S_AT, 214255_AT, 206163_AT, 214705_AT, 217047_S_AT, 208443_X_AT, 213943_AT, 218149_S_AT, 201010_S_AT, 205447_S_AT, 213823_AT, 209568_S_AT, 217738_AT, 221911_AT, 212276_AT, 206336_AT, 201369_S_AT, 213348_AT, 208378_X_AT, 204790_AT, 202036_S_AT, 218486_AT, 204363_AT, 210511_S_AT, 206363_AT, 205289_AT, 204011_AT, 218723_S_AT, 207574_S_AT, 204470_AT, 204131_S_AT, 39402_AT, 220109_AT, 203131_AT, 208394_X_AT, 218995_S_AT, 219187_AT, 205117_AT, 209815_AT, 204422_S_AT, 200749_AT, 202628_S_AT, 203879_AT, 206631_AT, 206987_X_AT, 205253_AT, 210135_S_AT, 212614_AT, 206373_AT, 201976_S_AT, 219257_S_AT, 201631_S_AT, 204396_S_AT, 202951_AT, 213568_AT, 202627_S_AT, 210310_S_AT, 215086_AT, 204731_AT, 205290_S_AT

GO:0007399	nervous system development	19	17.924528	2.62E-04	204470_AT, 209120_AT, 214111_AT, 210302_S_AT, 205253_AT, 209440_AT, 203131_AT, 208447_S_AT, 210135_S_AT, 219187_AT, 205117_AT, 213348_AT, 208378_X_AT, 206373_AT, 209815_AT, 204422_S_AT, 210310_S_AT, 220319_S_AT, 210511_S_AT, 208443_X_AT, 213943_AT, 205289_AT, 205290_S_AT
GO:0045165	cell fate commitment	7	6.6037736	3.27E-04	205117_AT, 204011_AT, 213823_AT, 221841_S_AT, 209120_AT, 204422_S_AT, 205289_AT, 205290_S_AT
GO:0007389	pattern specification process	9	8.490566	4.03E-04	206373_AT, 213823_AT, 202036_S_AT, 213150_AT, 209815_AT, 209120_AT, 204463_S_AT, 205253_AT, 218995_S_AT
GO:0032355	response to estradiol stimulus	5	4.7169811	4.52E-04	39402_AT, 209815_AT, 203131_AT, 201010_S_AT, 201041_S_AT
GO:0001525	angiogenesis	7	6.6037736	4.58E-04	205117_AT, 39402_AT, 204422_S_AT, 204463_S_AT, 206987_X_AT, 204195_S_AT, 218995_S_AT
GO:0006468	protein amino acid phosphorylation	14	13.207547	4.59E-04	205447_S_AT, 207574_S_AT, 203879_AT, 39402_AT, 221840_AT, 203131_AT, 209304_X_AT, 205265_S_AT, 215664_S_AT, 204790_AT, 204396_S_AT, 202951_AT, 204422_S_AT, 204731_AT, 205290_S_AT, 205289_AT
GO:0045859	regulation of protein kinase activity	10	9.4339623	4.80E-04	204011_AT, 213348_AT, 218723_S_AT, 207574_S_AT, 39402_AT, 219257_S_AT, 201853_S_AT, 204422_S_AT, 204463_S_AT, 209304_X_AT, 218995_S_AT
GO:0030324	lung development	6	5.6603774	5.14E-04	205117_AT, 204011_AT, 221541_AT, 204422_S_AT, 206987_X_AT, 203131_AT

GO:0007242	intracellular signaling cascade	20	18.867925	5.34E-04	205447_S_AT, 207574_S_AT, 204470_AT, 203879_AT, 39402_AT, 209568_S_AT, 204463_S_AT, 209304_X_AT, 201041_S_AT, 218995_S_AT, 219187_AT, 205117_AT, 204790_AT, 201830_S_AT, 219257_S_AT, 202951_AT, 204396_S_AT, 204422_S_AT, 200749_AT, 214705_AT, 204731_AT
GO:0007507	heart development	8	7.5471698	5.75E-04	204790_AT, 209815_AT, 204463_S_AT, 209765_AT, 208443_X_AT, 204731_AT, 210135_S_AT, 218995_S_AT, 205289_AT, 205290_S_AT
GO:0030323	respiratory tube development	6	5.6603774	5.89E-04	205117_AT, 204011_AT, 221541_AT, 204422_S_AT, 206987_X_AT, 203131_AT
GO:0043549	regulation of kinase activity	10	9.4339623	6.14E-04	204011_AT, 213348_AT, 218723_S_AT, 207574_S_AT, 39402_AT, 219257_S_AT, 201853_S_AT, 204422_S_AT, 204463_S_AT, 209304_X_AT, 218995_S_AT
GO:0048736	appendage development	6	5.6603774	6.16E-04	213823_AT, 213150_AT, 209815_AT, 209120_AT, 205253_AT, 213943_AT
GO:0060173	limb development	6	5.6603774	6.16E-04	213823_AT, 213150_AT, 209815_AT, 209120_AT, 205253_AT, 213943_AT
GO:0008285	negative regulation of cell proliferation	10	9.4339623	6.65E-04	213348_AT, 204470_AT, 39402_AT, 218486_AT, 221841_S_AT, 209815_AT, 204422_S_AT, 205265_S_AT, 204731_AT, 205289_AT, 205290_S_AT
GO:0045766	positive regulation of angiogenesis	4	3.7735849	6.65E-04	39402_AT, 219257_S_AT, 204422_S_AT, 204363_AT
GO:0007169	transmembrane receptor protein tyrosine kinase signalin	8	7.5471698	7.33E-04	205117_AT, 215664_S_AT, 208378_X_AT, 212614_AT, 204422_S_AT, 210310_S_AT, 206987_X_AT, 203131_AT, 201010_S_AT
GO:0060541	respiratory system development	6	5.6603774	7.64E-04	205117_AT, 204011_AT, 221541_AT, 204422_S_AT, 206987_X_AT, 203131_AT

GO:0016310	phosphorylation	15	14.150943	7.99E-04	205447_S_AT, 207574_S_AT, 203879_AT, 39402_AT, 221840_AT, 215850_S_AT, 203131_AT, 209304_X_AT, 205265_S_AT, 215664_S_AT, 204790_AT, 202951_AT, 204396_S_AT, 204422_S_AT, 204731_AT, 205290_S_AT, 205289_AT
GO:0045765	regulation of angiogenesis	5	4.7169811	8.13E-04	39402_AT, 219257_S_AT, 204422_S_AT, 204363_AT, 202627_S_AT, 202628_S_AT
GO:0051338	regulation of transferase activity	10	9.4339623	8.23E-04	204011_AT, 213348_AT, 218723_S_AT, 207574_S_AT, 39402_AT, 219257_S_AT, 201853_S_AT, 204422_S_AT, 204463_S_AT, 209304_X_AT, 218995_S_AT
GO:0030334	regulation of cell migration	7	6.6037736	9.20E-04	204790_AT, 219257_S_AT, 204422_S_AT, 204363_AT, 203131_AT, 204731_AT, 218995_S_AT
GO:0008543	fibroblast growth factor receptor signaling pathway	4	3.7735849	9.22E-04	205117_AT, 208378_X_AT, 204422_S_AT, 210310_S_AT, 206987_X_AT
GO:0033273	response to vitamin	5	4.7169811	9.69E-04	39402_AT, 221841_S_AT, 209815_AT, 201041_S_AT, 205289_AT, 205290_S_AT
GO:0032844	regulation of homeostatic process	6	5.6603774	9.75E-04	204790_AT, 204131_S_AT, 204463_S_AT, 220109_AT, 215086_AT, 210511_S_AT
GO:0060562	epithelial tube morphogenesis	5	4.7169811	0.0010257	213823_AT, 209815_AT, 205253_AT, 213943_AT, 205289_AT, 205290_S_AT
GO:0001932	regulation of protein amino acid phosphorylation	7	6.6037736	0.0010382	204790_AT, 39402_AT, 204422_S_AT, 204463_S_AT, 215086_AT, 218995_S_AT, 205289_AT, 205290_S_AT

GO:0048523	negative regulation of cellular process	23	21.698113	0.0011016	204470_AT, 39402_AT, 221841_S_AT, 209120_AT, 204463_S_AT, 205253_AT, 205265_S_AT, 218995_S_AT, 213348_AT, 212614_AT, 204790_AT, 202036_S_AT, 218486_AT, 209815_AT, 219257_S_AT, 201631_S_AT, 204422_S_AT, 204363_AT, 210511_S_AT, 215086_AT, 213943_AT, 204731_AT, 205290_S_AT, 205289_AT
GO:0051173	positive regulation of nitrogen compound metabolic proc	13	12.264151	0.0011393	204131_S_AT, 39402_AT, 221841_S_AT, 209120_AT, 205253_AT, 204195_S_AT, 203131_AT, 218995_S_AT, 209348_S_AT, 204422_S_AT, 200749_AT, 210511_S_AT, 206363_AT, 205290_S_AT, 205289_AT
GO:0031327	negative regulation of cellular biosynthetic process	12	11.320755	0.0012318	221841_S_AT, 209120_AT, 204463_S_AT, 218995_S_AT, 213348_AT, 204790_AT, 212614_AT, 218486_AT, 204422_S_AT, 210511_S_AT, 213943_AT, 205290_S_AT, 205289_AT
GO:0032526	response to retinoic acid	4	3.7735849	0.00135	221841_S_AT, 209815_AT, 201041_S_AT, 205289_AT, 205290_S_AT
GO:0040007	growth	7	6.6037736	0.0013861	212614_AT, 213823_AT, 204131_S_AT, 202627_S_AT, 210511_S_AT, 202628_S_AT, 204731_AT, 205289_AT, 205290_S_AT
GO:0009890	negative regulation of biosynthetic process	12	11.320755	0.0014593	221841_S_AT, 209120_AT, 204463_S_AT, 218995_S_AT, 213348_AT, 204790_AT, 212614_AT, 218486_AT, 204422_S_AT, 210511_S_AT, 213943_AT, 205290_S_AT, 205289_AT

GO:0048519	negative regulation of biological process	24	22.641509	0.001489	204470_AT, 39402_AT, 221841_S_AT, 209120_AT, 204463_S_AT, 205265_S_AT, 218995_S_AT, 209815_AT, 204422_S_AT, 213943_AT, 202628_S_AT, 205253_AT, 213348_AT, 204790_AT, 212614_AT, 218486_AT, 202036_S_AT, 201631_S_AT, 219257_S_AT, 202627_S_AT, 204363_AT, 215086_AT, 210511_S_AT, 205289_AT, 205290_S_AT, 204731_AT
GO:0043009	chordate embryonic development	9	8.490566	0.0016248	202036_S_AT, 209815_AT, 213568_AT, 204463_S_AT, 205253_AT, 213943_AT, 203131_AT, 204731_AT, 218995_S_AT
GO:0051726	regulation of cell cycle	9	8.490566	0.0016248	213348_AT, 218723_S_AT, 203558_AT, 207574_S_AT, 39402_AT, 219257_S_AT, 210511_S_AT, 209304_X_AT, 218995_S_AT, 205289_AT, 205290_S_AT
GO:0009792	embryonic development ending in birth or egg hatching	9	8.490566	0.0017197	202036_S_AT, 209815_AT, 213568_AT, 204463_S_AT, 205253_AT, 213943_AT, 203131_AT, 204731_AT, 218995_S_AT
GO:0040012	regulation of locomotion	7	6.6037736	0.0017696	204790_AT, 219257_S_AT, 204422_S_AT, 204363_AT, 203131_AT, 204731_AT, 218995_S_AT
GO:0051270	regulation of cell motion	7	6.6037736	0.0018167	204790_AT, 219257_S_AT, 204422_S_AT, 204363_AT, 203131_AT, 204731_AT, 218995_S_AT
GO:0031328	positive regulation of cellular biosynthetic process	13	12.264151	0.001922	204131_S_AT, 39402_AT, 221841_S_AT, 209120_AT, 205253_AT, 204195_S_AT, 203131_AT, 218995_S_AT, 209348_S_AT, 204422_S_AT, 200749_AT, 210511_S_AT, 206363_AT, 205290_S_AT, 205289_AT

GO:0009892	negative regulation of metabolic process	14	13.207547	0.0019285	39402_AT, 221841_S_AT, 209120_AT, 204463_S_AT, 218995_S_AT, 213348_AT, 212614_AT, 204790_AT, 218486_AT, 204422_S_AT, 215086_AT, 210511_S_AT, 213943_AT, 205290_S_AT, 205289_AT
GO:0009966	regulation of signal transduction	15	14.150943	0.0019401	204011_AT, 39402_AT, 209568_S_AT, 206987_X_AT, 218995_S_AT, 213348_AT, 204790_AT, 206373_AT, 201830_S_AT, 209815_AT, 204396_S_AT, 204363_AT, 204422_S_AT, 210511_S_AT, 205290_S_AT, 205289_AT
GO:0031325	positive regulation of cellular metabolic process	15	14.150943	0.0019816	204131_S_AT, 39402_AT, 221841_S_AT, 209120_AT, 204463_S_AT, 205253_AT, 204195_S_AT, 203131_AT, 218995_S_AT, 204790_AT, 209348_S_AT, 204422_S_AT, 200749_AT, 210511_S_AT, 206363_AT, 205290_S_AT, 205289_AT
GO:0003002	regionalization	7	6.6037736	0.0020149	213823_AT, 202036_S_AT, 213150_AT, 209815_AT, 209120_AT, 205253_AT, 218995_S_AT
GO:0007267	cell-cell signaling	12	11.320755	0.002099	204011_AT, 213823_AT, 39402_AT, 217738_AT, 206336_AT, 206987_X_AT, 218995_S_AT, 205117_AT, 208378_X_AT, 204422_S_AT, 210310_S_AT, 210511_S_AT, 205290_S_AT, 205289_AT
GO:0045934	negative regulation of nucleobase, nucleoside, nucleotide	11	10.377358	0.0021282	213348_AT, 212614_AT, 204790_AT, 218486_AT, 221841_S_AT, 209120_AT, 204422_S_AT, 204463_S_AT, 213943_AT, 218995_S_AT, 205289_AT, 205290_S_AT
GO:0009891	positive regulation of biosynthetic process	13	12.264151	0.0021685	204131_S_AT, 39402_AT, 221841_S_AT, 209120_AT, 205253_AT, 204195_S_AT, 203131_AT, 218995_S_AT, 209348_S_AT, 204422_S_AT, 200749_AT, 210511_S_AT, 206363_AT, 205290_S_AT, 205289_AT

GO:0051781	positive regulation of cell division	4	3.7735849	0.002197	205117_AT, 208378_X_AT, 39402_AT, 204422_S_AT, 210310_S_AT
GO:0051172	negative regulation of nitrogen compound metabolic pro	11	10.377358	0.0023494	213348_AT, 212614_AT, 204790_AT, 218486_AT, 221841_S_AT, 209120_AT, 204422_S_AT, 204463_S_AT, 213943_AT, 218995_S_AT, 205289_AT, 205290_S_AT
GO:0060284	regulation of cell development	7	6.6037736	0.0024598	204790_AT, 213823_AT, 204422_S_AT, 205253_AT, 204731_AT, 218995_S_AT, 205289_AT, 205290_S_AT
GO:0051094	positive regulation of developmental process	8	7.5471698	0.002536	39402_AT, 213823_AT, 204131_S_AT, 219257_S_AT, 204422_S_AT, 204363_AT, 210511_S_AT, 205289_AT, 205290_S_AT
GO:0045892	negative regulation of transcription, DNA-dependent	9	8.490566	0.0025573	213348_AT, 212614_AT, 204790_AT, 218486_AT, 221841_S_AT, 209120_AT, 204422_S_AT, 213943_AT, 205289_AT, 205290_S_AT
GO:0035113	embryonic appendage morphogenesis	5	4.7169811	0.0026924	213823_AT, 213150_AT, 209815_AT, 205253_AT, 213943_AT
GO:0030326	embryonic limb morphogenesis	5	4.7169811	0.0026924	213823_AT, 213150_AT, 209815_AT, 205253_AT, 213943_AT
GO:0033189	response to vitamin A	4	3.7735849	0.0027203	221841_S_AT, 209815_AT, 201041_S_AT, 205289_AT, 205290_S_AT
GO:0060389	pathway-restricted SMAD protein phosphorylation	3	2.8301887	0.0027726	204790_AT, 204731_AT, 205289_AT, 205290_S_AT
GO:0051253	negative regulation of RNA metabolic process	9	8.490566	0.0028337	213348_AT, 212614_AT, 204790_AT, 218486_AT, 221841_S_AT, 209120_AT, 204422_S_AT, 213943_AT, 205289_AT, 205290_S_AT
GO:0031324	negative regulation of cellular metabolic process	13	12.264151	0.0029005	221841_S_AT, 209120_AT, 204463_S_AT, 218995_S_AT, 213348_AT, 212614_AT, 204790_AT, 218486_AT, 204422_S_AT, 215086_AT, 210511_S_AT, 213943_AT, 205290_S_AT, 205289_AT

GO:0030335	positive regulation of cell migration	5	4.7169811	0.0029237	219257_S_AT, 204422_S_AT, 204363_AT, 203131_AT, 218995_S_AT
GO:0009893	positive regulation of metabolic process	15	14.150943	0.0030104	204131_S_AT, 39402_AT, 221841_S_AT, 209120_AT, 204463_S_AT, 205253_AT, 204195_S_AT, 203131_AT, 218995_S_AT, 204790_AT, 209348_S_AT, 204422_S_AT, 200749_AT, 210511_S_AT, 206363_AT, 205290_S_AT, 205289_AT
GO:0006357	regulation of transcription from RNA polymerase II prom	13	12.264151	0.0031382	204131_S_AT, 221841_S_AT, 209120_AT, 205253_AT, 204195_S_AT, 213348_AT, 204790_AT, 218486_AT, 209348_S_AT, 204422_S_AT, 210511_S_AT, 213943_AT, 206363_AT, 205290_S_AT, 205289_AT
GO:0007243	protein kinase cascade	9	8.490566	0.0032382	219187_AT, 205117_AT, 205447_S_AT, 207574_S_AT, 39402_AT, 202951_AT, 204422_S_AT, 209304_X_AT, 204731_AT, 218995_S_AT
GO:0001759	induction of an organ	3	2.8301887	0.0032626	205117_AT, 213823_AT, 204422_S_AT
GO:0042326	negative regulation of phosphorylation	4	3.7735849	0.0033144	213348_AT, 204790_AT, 215086_AT, 210511_S_AT
GO:0010558	negative regulation of macromolecule biosynthetic proce	11	10.377358	0.0034269	213348_AT, 212614_AT, 204790_AT, 218486_AT, 221841_S_AT, 209120_AT, 204422_S_AT, 210511_S_AT, 213943_AT, 218995_S_AT, 205289_AT, 205290_S_AT
GO:0010646	regulation of cell communication	16	15.09434	0.0034384	204011_AT, 39402_AT, 209568_S_AT, 206987_X_AT, 220109_AT, 218995_S_AT, 213348_AT, 204790_AT, 206373_AT, 201830_S_AT, 209815_AT, 204396_S_AT, 204363_AT, 204422_S_AT, 210511_S_AT, 205290_S_AT, 205289_AT

GO:0048468	cell development	12	11.320755	0.0036189	204131_S_AT, 221841_S_AT, 201853_S_AT, 204463_S_AT, 214111_AT, 206987_X_AT, 218995_S_AT, 213348_AT, 209348_S_AT, 200749_AT, 206363_AT, 204731_AT, 205290_S_AT, 205289_AT
GO:0045860	positive regulation of protein kinase activity	7	6.6037736	0.0037281	207574_S_AT, 39402_AT, 219257_S_AT, 201853_S_AT, 204422_S_AT, 204463_S_AT, 209304_X_AT, 218995_S_AT
GO:0051302	regulation of cell division	4	3.7735849	0.0037513	205117_AT, 208378_X_AT, 39402_AT, 204422_S_AT, 210310_S_AT
GO:0010171	body morphogenesis	3	2.8301887	0.0037899	212614_AT, 210302_S_AT, 203131_AT
GO:0045936	negative regulation of phosphate metabolic process	4	3.7735849	0.0039823	213348_AT, 204790_AT, 215086_AT, 210511_S_AT
GO:0010563	negative regulation of phosphorus metabolic process	4	3.7735849	0.0039823	213348_AT, 204790_AT, 215086_AT, 210511_S_AT
GO:0010557	positive regulation of macromolecule biosynthetic proces	12	11.320755	0.0040651	204131_S_AT, 39402_AT, 221841_S_AT, 209120_AT, 205253_AT, 204195_S_AT, 203131_AT, 209348_S_AT, 204422_S_AT, 200749_AT, 210511_S_AT, 206363_AT, 205290_S_AT, 205289_AT
GO:0060429	epithelium development	7	6.6037736	0.0040659	213823_AT, 209815_AT, 209120_AT, 205253_AT, 213943_AT, 201010_S_AT, 205289_AT, 205290_S_AT
GO:0042592	homeostatic process	13	12.264151	0.0040764	204131_S_AT, 39402_AT, 204463_S_AT, 201479_AT, 220109_AT, 204195_S_AT, 218995_S_AT, 204790_AT, 209815_AT, 202627_S_AT, 215086_AT, 210511_S_AT, 202628_S_AT, 204731_AT
GO:0040017	positive regulation of locomotion	5	4.7169811	0.0041337	219257_S_AT, 204422_S_AT, 204363_AT, 203131_AT, 218995_S_AT
GO:0051272	positive regulation of cell motion	5	4.7169811	0.0041337	219257_S_AT, 204422_S_AT, 204363_AT, 203131_AT, 218995_S_AT

GO:0035107	appendage morphogenesis	5	4.7169811	0.0042861	213823_AT, 213150_AT, 209815_AT, 205253_AT, 213943_AT
GO:0035108	limb morphogenesis	5	4.7169811	0.0042861	213823_AT, 213150_AT, 209815_AT, 205253_AT, 213943_AT
GO:0010604	positive regulation of macromolecule metabolic process	14	13.207547	0.0043442	204131_S_AT, 39402_AT, 221841_S_AT, 209120_AT, 204463_S_AT, 205253_AT, 204195_S_AT, 203131_AT, 204790_AT, 209348_S_AT, 204422_S_AT, 200749_AT, 210511_S_AT, 206363_AT, 205290_S_AT, 205289_AT
GO:0042221	response to chemical stimulus	18	16.981132	0.0043787	204470_AT, 39402_AT, 221841_S_AT, 206336_AT, 212276_AT, 204463_S_AT, 220109_AT, 203131_AT, 201041_S_AT, 218995_S_AT, 209815_AT, 202085_AT, 204396_S_AT, 204422_S_AT, 202627_S_AT, 202628_S_AT, 204731_AT, 201010_S_AT, 205290_S_AT, 205289_AT
GO:0033674	positive regulation of kinase activity	7	6.6037736	0.004426	207574_S_AT, 39402_AT, 219257_S_AT, 201853_S_AT, 204422_S_AT, 204463_S_AT, 209304_X_AT, 218995_S_AT
GO:0045893	positive regulation of transcription, DNA-dependent	10	9.4339623	0.004459	204131_S_AT, 221841_S_AT, 209348_S_AT, 209120_AT, 204422_S_AT, 210511_S_AT, 205253_AT, 200749_AT, 204195_S_AT, 206363_AT, 205289_AT, 205290_S_AT
GO:0002009	morphogenesis of an epithelium	5	4.7169811	0.0046022	213823_AT, 209815_AT, 205253_AT, 213943_AT, 205289_AT, 205290_S_AT
GO:0051254	positive regulation of RNA metabolic process	10	9.4339623	0.0047069	204131_S_AT, 221841_S_AT, 209348_S_AT, 209120_AT, 204422_S_AT, 210511_S_AT, 205253_AT, 200749_AT, 204195_S_AT, 206363_AT, 205289_AT, 205290_S_AT

GO:0048762	mesenchymal cell differentiation	4	3.7735849	0.0047265	204463_S_AT, 204731_AT, 218995_S_AT, 205289_AT, 205290_S_AT
GO:0014031	mesenchymal cell development	4	3.7735849	0.0047265	204463_S_AT, 204731_AT, 218995_S_AT, 205289_AT, 205290_S_AT
GO:0065009	regulation of molecular function	15	14.150943	0.0047392	204011_AT, 218723_S_AT, 207574_S_AT, 39402_AT, 201853_S_AT, 209120_AT, 204463_S_AT, 205253_AT, 209304_X_AT, 218995_S_AT, 213348_AT, 204790_AT, 219257_S_AT, 204422_S_AT, 204363_AT, 204731_AT
GO:0043687	post-translational protein modification	17	16.037736	0.0047493	205447_S_AT, 207574_S_AT, 203879_AT, 39402_AT, 221840_AT, 201853_S_AT, 203131_AT, 209304_X_AT, 205265_S_AT, 201041_S_AT, 215664_S_AT, 204790_AT, 202951_AT, 204396_S_AT, 204422_S_AT, 220319_S_AT, 204731_AT, 205290_S_AT, 205289_AT
GO:0051090	regulation of transcription factor activity	5	4.7169811	0.0049337	204790_AT, 39402_AT, 209120_AT, 205253_AT, 204731_AT
GO:0060485	mesenchyme development	4	3.7735849	0.0049919	204463_S_AT, 204731_AT, 218995_S_AT, 205289_AT, 205290_S_AT
GO:0043627	response to estrogen stimulus	5	4.7169811	0.0052809	39402_AT, 209815_AT, 203131_AT, 201010_S_AT, 201041_S_AT
GO:0051347	positive regulation of transferase activity	7	6.6037736	0.0053218	207574_S_AT, 39402_AT, 219257_S_AT, 201853_S_AT, 204422_S_AT, 204463_S_AT, 209304_X_AT, 218995_S_AT
GO:0043010	camera-type eye development	5	4.7169811	0.0056441	221841_S_AT, 209348_S_AT, 210302_S_AT, 206163_AT, 204195_S_AT, 206363_AT
GO:0051240	positive regulation of multicellular organismal process	7	6.6037736	0.0057599	39402_AT, 219257_S_AT, 204422_S_AT, 204363_AT, 220109_AT, 218995_S_AT, 205289_AT, 205290_S_AT

GO:0001655	urogenital system development	5	4.7169811	0.0062196	212614_AT, 213823_AT, 202036_S_AT, 205253_AT, 205289_AT, 205290_S_AT
GO:0048705	skeletal system morphogenesis	5	4.7169811	0.0066242	212614_AT, 213823_AT, 213568_AT, 206987_X_AT, 203131_AT
GO:0031128	developmental induction	3	2.8301887	0.0069693	205117_AT, 213823_AT, 204422_S_AT
GO:0045168	cell-cell signaling involved in cell fate specification	3	2.8301887	0.0069693	205117_AT, 213823_AT, 204422_S_AT
GO:0048008	platelet-derived growth factor receptor signaling pathwa	3	2.8301887	0.0069693	212614_AT, 203131_AT, 201010_S_AT
GO:0032147	activation of protein kinase activity	5	4.7169811	0.007046	207574_S_AT, 219257_S_AT, 204422_S_AT, 204463_S_AT, 209304_X_AT, 218995_S_AT
GO:0032879	regulation of localization	11	10.377358	0.0072979	204790_AT, 39402_AT, 219257_S_AT, 204422_S_AT, 204363_AT, 204463_S_AT, 215086_AT, 210511_S_AT, 203131_AT, 204731_AT, 218995_S_AT
GO:0051252	regulation of RNA metabolic process	22	20.754717	0.0073261	204131_S_AT, 208025_S_AT, 221841_S_AT, 209120_AT, 204195_S_AT, 213150_AT, 209348_S_AT, 204422_S_AT, 208443_X_AT, 200749_AT, 213943_AT, 218149_S_AT, 213823_AT, 221911_AT, 205253_AT, 201369_S_AT, 210135_S_AT, 213348_AT, 204790_AT, 212614_AT, 218486_AT, 210511_S_AT, 206363_AT, 205289_AT, 205290_S_AT
GO:0009605	response to external stimulus	14	13.207547	0.0073887	204470_AT, 39402_AT, 221841_S_AT, 206336_AT, 220109_AT, 203131_AT, 201041_S_AT, 209815_AT, 202627_S_AT, 204422_S_AT, 204363_AT, 210511_S_AT, 202628_S_AT, 201010_S_AT, 205290_S_AT, 205289_AT
GO:0003006	reproductive developmental process	7	6.6037736	0.0080642	204131_S_AT, 202036_S_AT, 201853_S_AT, 210511_S_AT, 205253_AT, 200749_AT, 203131_AT

GO:0045935	positive regulation of nucleobase, nucleoside, nucleotide	11	10.377358	0.0085	204131_S_AT, 221841_S_AT, 209120_AT, 205253_AT, 204195_S_AT, 203131_AT, 209348_S_AT, 204422_S_AT, 200749_AT, 210511_S_AT, 206363_AT, 205290_S_AT, 205289_AT
GO:0051101	regulation of DNA binding	5	4.7169811	0.0086609	204790_AT, 39402_AT, 209120_AT, 205253_AT, 204731_AT
GO:0007165	signal transduction	30	28.301887	0.0088477	204011_AT, 204470_AT, 207574_S_AT, 39402_AT, 209120_AT, 204463_S_AT, 209304_X_AT, 218995_S_AT, 201041_S_AT, 219187_AT, 205117_AT, 201830_S_AT, 209815_AT, 204422_S_AT, 200749_AT, 214705_AT, 217047_S_AT, 205447_S_AT, 203879_AT, 209568_S_AT, 217738_AT, 206631_AT, 206336_AT, 206987_X_AT, 204790_AT, 202036_S_AT, 201976_S_AT, 219257_S_AT, 202951_AT, 204396_S_AT, 204731_AT
GO:0060675	ureteric bud morphogenesis	3	2.8301887	0.0092935	213823_AT, 205253_AT, 205289_AT, 205290_S_AT
GO:0001658	branching involved in ureteric bud morphogenesis	3	2.8301887	0.0092935	213823_AT, 205253_AT, 205289_AT, 205290_S_AT
GO:0010605	negative regulation of macromolecule metabolic process	12	11.320755	0.0094384	221841_S_AT, 209120_AT, 218995_S_AT, 213348_AT, 204790_AT, 212614_AT, 218486_AT, 204422_S_AT, 210511_S_AT, 215086_AT, 213943_AT, 205290_S_AT, 205289_AT

GO:0007166	cell surface receptor linked signal transduction	22	20.754717	0.0095122	204470_AT, 221840_AT, 206631_AT, 206336_AT, 204463_S_AT, 206987_X_AT, 203131_AT, 218995_S_AT, 205117_AT, 215664_S_AT, 208378_X_AT, 212614_AT, 204790_AT, 202036_S_AT, 209815_AT, 219257_S_AT, 204396_S_AT, 204422_S_AT, 210310_S_AT, 210511_S_AT, 204731_AT, 201010_S_AT, 205290_S_AT, 205289_AT
GO:0009954	proximal/distal pattern formation	3	2.8301887	0.0101349	213823_AT, 213150_AT, 205253_AT
GO:0016481	negative regulation of transcription	9	8.490566	0.0114174	213348_AT, 212614_AT, 204790_AT, 218486_AT, 221841_S_AT, 209120_AT, 204422_S_AT, 213943_AT, 205289_AT, 205290_S_AT
GO:0001654	eye development	5	4.7169811	0.0116581	221841_S_AT, 209348_S_AT, 210302_S_AT, 206163_AT, 204195_S_AT, 206363_AT
GO:0006355	regulation of transcription, DNA-dependent	21	19.811321	0.0118482	213823_AT, 204131_S_AT, 208025_S_AT, 221841_S_AT, 221911_AT, 209120_AT, 205253_AT, 204195_S_AT, 210135_S_AT, 213348_AT, 212614_AT, 204790_AT, 218486_AT, 213150_AT, 209348_S_AT, 204422_S_AT, 210511_S_AT, 200749_AT, 208443_X_AT, 206363_AT, 213943_AT, 218149_S_AT, 205290_S_AT, 205289_AT
GO:0045944	positive regulation of transcription from RNA polymerase	8	7.5471698	0.0118628	204131_S_AT, 221841_S_AT, 209348_S_AT, 204422_S_AT, 210511_S_AT, 205253_AT, 204195_S_AT, 206363_AT, 205289_AT, 205290_S_AT
GO:0050680	negative regulation of epithelial cell proliferation	3	2.8301887	0.0119145	213348_AT, 209815_AT, 204731_AT
GO:0003007	heart morphogenesis	4	3.7735849	0.0127104	204790_AT, 209815_AT, 204731_AT, 205289_AT, 205290_S_AT

GO:0045941	positive regulation of transcription	10	9.4339623	0.0127253	204131_S_AT, 221841_S_AT, 209348_S_AT, 209120_AT, 204422_S_AT, 210511_S_AT, 205253_AT, 200749_AT, 204195_S_AT, 206363_AT, 205289_AT, 205290_S_AT
GO:0046888	negative regulation of hormone secretion	3	2.8301887	0.0128519	39402_AT, 210511_S_AT, 218995_S_AT
GO:0051216	cartilage development	4	3.7735849	0.0131846	213823_AT, 206987_X_AT, 218995_S_AT, 205289_AT, 205290_S_AT
GO:0010719	negative regulation of epithelial to mesenchymal transitic	2	1.8867925	0.0132743	204790_AT, 204731_AT
GO:0031323	regulation of cellular metabolic process	34	32.075472	0.0138728	204011_AT, 218723_S_AT, 207574_S_AT, 39402_AT, 204131_S_AT, 208025_S_AT, 221841_S_AT, 209120_AT, 201853_S_AT, 204463_S_AT, 204195_S_AT, 203131_AT, 209304_X_AT, 218995_S_AT, 213150_AT, 209348_S_AT, 204422_S_AT, 208443_X_AT, 200749_AT, 213943_AT, 218149_S_AT, 201010_S_AT, 213823_AT, 221911_AT, 205253_AT, 201369_S_AT, 210135_S_AT, 213348_AT, 212614_AT, 204790_AT, 218486_AT, 219257_S_AT, 210511_S_AT, 215086_AT, 206363_AT, 204731_AT, 205290_S_AT, 205289_AT
GO:0031399	regulation of protein modification process	7	6.6037736	0.0139054	204790_AT, 39402_AT, 204422_S_AT, 204463_S_AT, 215086_AT, 218995_S_AT, 205289_AT, 205290_S_AT
GO:0007584	response to nutrient	5	4.7169811	0.0142111	39402_AT, 221841_S_AT, 209815_AT, 201041_S_AT, 205289_AT, 205290_S_AT
GO:0009952	anterior/posterior pattern formation	5	4.7169811	0.0142111	213823_AT, 202036_S_AT, 213150_AT, 209120_AT, 205253_AT

GO:0006950	response to stress	20	18.867925	0.0142974	216191_S_AT, 205447_S_AT, 207574_S_AT, 204470_AT, 204131_S_AT, 39402_AT, 206336_AT, 204463_S_AT, 220109_AT, 203131_AT, 208070_S_AT, 209304_X_AT, 201041_S_AT, 218995_S_AT, 204790_AT, 204422_S_AT, 204363_AT, 202627_S_AT, 210511_S_AT, 202628_S_AT, 201010_S_AT, 205290_S_AT, 205289_AT
GO:0045596	negative regulation of cell differentiation	6	5.6603774	0.0146457	204790_AT, 221841_S_AT, 210511_S_AT, 205253_AT, 213943_AT, 204731_AT
GO:0010628	positive regulation of gene expression	10	9.4339623	0.0151937	204131_S_AT, 221841_S_AT, 209348_S_AT, 209120_AT, 204422_S_AT, 210511_S_AT, 205253_AT, 200749_AT, 204195_S_AT, 206363_AT, 205289_AT, 205290_S_AT
GO:0007154	cell communication	12	11.320755	0.0163841	204011_AT, 213823_AT, 39402_AT, 217738_AT, 206336_AT, 206987_X_AT, 218995_S_AT, 205117_AT, 208378_X_AT, 204422_S_AT, 210310_S_AT, 210511_S_AT, 205290_S_AT, 205289_AT
GO:0010629	negative regulation of gene expression	9	8.490566	0.0190563	213348_AT, 212614_AT, 204790_AT, 218486_AT, 221841_S_AT, 209120_AT, 204422_S_AT, 213943_AT, 205289_AT, 205290_S_AT
GO:0051098	regulation of binding	5	4.7169811	0.0190693	204790_AT, 39402_AT, 209120_AT, 205253_AT, 204731_AT
GO:0060021	palate development	3	2.8301887	0.0191148	212614_AT, 203131_AT, 204731_AT
GO:0010771	negative regulation of cell morphogenesis involved in diff	2	1.8867925	0.019846	204790_AT, 204731_AT
GO:0060317	cardiac epithelial to mesenchymal transition	2	1.8867925	0.019846	204731_AT, 205289_AT, 205290_S_AT
GO:0048878	chemical homeostasis	9	8.490566	0.0207262	204790_AT, 39402_AT, 204131_S_AT, 209815_AT, 204463_S_AT, 202627_S_AT, 220109_AT, 215086_AT, 202628_S_AT, 218995_S_AT

GO:0001934	positive regulation of protein amino acid phosphorylatior	4	3.7735849	0.0215127	39402_AT, 204422_S_AT, 204463_S_AT, 205289_AT, 205290_S_AT
GO:0033673	negative regulation of kinase activity	4	3.7735849	0.0221496	204011_AT, 213348_AT, 207574_S_AT, 39402_AT, 209304_X_AT
GO:0001657	ureteric bud development	3	2.8301887	0.022637	213823_AT, 205253_AT, 205289_AT, 205290_S_AT
GO:0043412	biopolymer modification	18	16.981132	0.0228386	205447_S_AT, 207574_S_AT, 203879_AT, 39402_AT, 221840_AT, 201853_S_AT, 201479_AT, 203131_AT, 209304_X_AT, 205265_S_AT, 201041_S_AT, 215664_S_AT, 204790_AT, 202951_AT, 204396_S_AT, 204422_S_AT, 220319_S_AT, 204731_AT, 205290_S_AT, 205289_AT
GO:0007205	activation of protein kinase C activity by G-protein couple	3	2.8301887	0.0238662	219257_S_AT, 204463_S_AT, 218995_S_AT
GO:0030193	regulation of blood coagulation	3	2.8301887	0.0238662	204363_AT, 202627_S_AT, 202628_S_AT, 218995_S_AT
GO:0050790	regulation of catalytic activity	12	11.320755	0.0246993	204011_AT, 207574_S_AT, 218723_S_AT, 39402_AT, 201853_S_AT, 204463_S_AT, 209304_X_AT, 218995_S_AT, 213348_AT, 204790_AT, 219257_S_AT, 204363_AT, 204422_S_AT
GO:0040011	locomotion	8	7.5471698	0.0248318	212614_AT, 204470_AT, 39402_AT, 209120_AT, 204422_S_AT, 206336_AT, 213943_AT, 204731_AT
GO:0009611	response to wounding	9	8.490566	0.0248616	204470_AT, 39402_AT, 204422_S_AT, 204363_AT, 206336_AT, 202627_S_AT, 220109_AT, 203131_AT, 202628_S_AT, 205289_AT, 205290_S_AT
GO:0043583	ear development	4	3.7735849	0.0254875	204011_AT, 206373_AT, 203131_AT, 218995_S_AT

GO:0019219	regulation of nucleobase, nucleoside, nucleotide and nuc	28	26.415094	0.0257783	39402_AT, 204131_S_AT, 208025_S_AT, 221841_S_AT, 209120_AT, 204463_S_AT, 204195_S_AT, 203131_AT, 218995_S_AT, 213150_AT, 209348_S_AT, 204422_S_AT, 208443_X_AT, 200749_AT, 213943_AT, 201010_S_AT, 218149_S_AT, 213823_AT, 221911_AT, 205253_AT, 201369_S_AT, 210135_S_AT, 213348_AT, 212614_AT, 204790_AT, 218486_AT, 210511_S_AT, 206363_AT, 205289_AT, 205290_S_AT, 204731_AT
GO:0019222	regulation of metabolic process	34	32.075472	0.0261085	204011_AT, 218723_S_AT, 207574_S_AT, 39402_AT, 204131_S_AT, 208025_S_AT, 221841_S_AT, 209120_AT, 201853_S_AT, 204463_S_AT, 204195_S_AT, 203131_AT, 209304_X_AT, 218995_S_AT, 213150_AT, 209348_S_AT, 204422_S_AT, 208443_X_AT, 200749_AT, 213943_AT, 218149_S_AT, 201010_S_AT, 213823_AT, 221911_AT, 205253_AT, 201369_S_AT, 210135_S_AT, 213348_AT, 212614_AT, 204790_AT, 218486_AT, 219257_S_AT, 210511_S_AT, 215086_AT, 206363_AT, 204731_AT, 205290_S_AT, 205289_AT
GO:0001822	kidney development	4	3.7735849	0.0261858	212614_AT, 213823_AT, 205253_AT, 205289_AT, 205290_S_AT
GO:0051348	negative regulation of transferase activity	4	3.7735849	0.0261858	204011_AT, 213348_AT, 207574_S_AT, 39402_AT, 209304_X_AT
GO:0040008	regulation of growth	7	6.6037736	0.0262554	201830_S_AT, 208025_S_AT, 209815_AT, 219257_S_AT, 204422_S_AT, 210511_S_AT, 208394_X_AT
GO:0014824	artery smooth muscle contraction	2	1.8867925	0.0263743	204463_S_AT, 218995_S_AT
GO:0014820	tonic smooth muscle contraction	2	1.8867925	0.0263743	204463_S_AT, 218995_S_AT

GO:0042327	positive regulation of phosphorylation	4	3.7735849	0.0268943	39402_AT, 204422_S_AT, 204463_S_AT, 205289_AT, 205290_S_AT
GO:0048568	embryonic organ development	5	4.7169811	0.0278343	204011_AT, 206373_AT, 213568_AT, 205253_AT, 218995_S_AT
GO:0051171	regulation of nitrogen compound metabolic process	28	26.415094	0.0286082	39402_AT, 204131_S_AT, 208025_S_AT, 221841_S_AT, 209120_AT, 204463_S_AT, 204195_S_AT, 203131_AT, 218995_S_AT, 213150_AT, 209348_S_AT, 204422_S_AT, 208443_X_AT, 200749_AT, 213943_AT, 201010_S_AT, 218149_S_AT, 213823_AT, 221911_AT, 205253_AT, 201369_S_AT, 210135_S_AT, 213348_AT, 212614_AT, 204790_AT, 218486_AT, 210511_S_AT, 206363_AT, 205289_AT, 205290_S_AT, 204731_AT
GO:0045937	positive regulation of phosphate metabolic process	4	3.7735849	0.0290812	39402_AT, 204422_S_AT, 204463_S_AT, 205289_AT, 205290_S_AT
GO:0010562	positive regulation of phosphorus metabolic process	4	3.7735849	0.0290812	39402_AT, 204422_S_AT, 204463_S_AT, 205289_AT, 205290_S_AT
GO:0051093	negative regulation of developmental process	6	5.6603774	0.0293466	204790_AT, 221841_S_AT, 210511_S_AT, 205253_AT, 213943_AT, 204731_AT
GO:0006464	protein modification process	17	16.037736	0.0298282	205447_S_AT, 207574_S_AT, 203879_AT, 39402_AT, 221840_AT, 201853_S_AT, 203131_AT, 209304_X_AT, 205265_S_AT, 201041_S_AT, 215664_S_AT, 204790_AT, 202951_AT, 204396_S_AT, 204422_S_AT, 220319_S_AT, 204731_AT, 205290_S_AT, 205289_AT
GO:0022414	reproductive process	11	10.377358	0.0300335	39402_AT, 204131_S_AT, 202036_S_AT, 213150_AT, 201853_S_AT, 203612_AT, 210511_S_AT, 205253_AT, 200749_AT, 203131_AT, 205289_AT, 205290_S_AT
GO:0050818	regulation of coagulation	3	2.8301887	0.0304068	204363_AT, 202627_S_AT, 202628_S_AT, 218995_S_AT

GO:0000003	reproduction	11	10.377358	0.0312191	39402_AT, 204131_S_AT, 202036_S_AT, 213150_AT, 201853_S_AT, 203612_AT, 210511_S_AT, 205253_AT, 200749_AT, 203131_AT, 205289_AT, 205290_S_AT
GO:0065008	regulation of biological quality	17	16.037736	0.0325821	204131_S_AT, 39402_AT, 204463_S_AT, 201479_AT, 220109_AT, 204195_S_AT, 218995_S_AT, 204790_AT, 209815_AT, 219257_S_AT, 214255_AT, 204422_S_AT, 204363_AT, 202627_S_AT, 210511_S_AT, 215086_AT, 202628_S_AT, 204731_AT
GO:0030218	erythrocyte differentiation	3	2.8301887	0.0332	210511_S_AT, 204195_S_AT, 204731_AT
GO:0030509	BMP signaling pathway	3	2.8301887	0.0346329	204790_AT, 204731_AT, 205289_AT, 205290_S_AT
GO:0001656	metanephros development	3	2.8301887	0.0346329	213823_AT, 205253_AT, 205289_AT, 205290_S_AT
GO:0009725	response to hormone stimulus	7	6.6037736	0.0357423	39402_AT, 209815_AT, 212276_AT, 203131_AT, 201010_S_AT, 204731_AT, 201041_S_AT
GO:0031401	positive regulation of protein modification process	5	4.7169811	0.0362079	204790_AT, 39402_AT, 204422_S_AT, 204463_S_AT, 205289_AT, 205290_S_AT
GO:0007204	elevation of cytosolic calcium ion concentration	4	3.7735849	0.0370306	39402_AT, 204463_S_AT, 215086_AT, 218995_S_AT
GO:0016477	cell migration	6	5.6603774	0.0370646	212614_AT, 39402_AT, 209120_AT, 204422_S_AT, 213943_AT, 204731_AT
GO:0048660	regulation of smooth muscle cell proliferation	3	2.8301887	0.0375696	219187_AT, 221841_S_AT, 218995_S_AT
GO:0042060	wound healing	5	4.7169811	0.0386617	39402_AT, 204422_S_AT, 204363_AT, 202627_S_AT, 203131_AT, 202628_S_AT
GO:0048545	response to steroid hormone stimulus	5	4.7169811	0.0392897	39402_AT, 209815_AT, 203131_AT, 201010_S_AT, 201041_S_AT
GO:0001558	regulation of cell growth	5	4.7169811	0.0405634	201830_S_AT, 219257_S_AT, 204422_S_AT, 210511_S_AT, 208394_X_AT
GO:0032582	negative regulation of gene-specific transcription	3	2.8301887	0.0405983	213348_AT, 204790_AT, 205289_AT, 205290_S_AT

GO:0044093	positive regulation of molecular function	9	8.490566	0.0413822	207574_S_AT, 39402_AT, 219257_S_AT, 201853_S_AT, 204422_S_AT, 204363_AT, 204463_S_AT, 209304_X_AT, 204731_AT, 218995_S_AT
GO:0042593	glucose homeostasis	3	2.8301887	0.0421462	204131_S_AT, 209815_AT, 202627_S_AT, 202628_S_AT
GO:0034101	erythrocyte homeostasis	3	2.8301887	0.0421462	210511_S_AT, 204195_S_AT, 204731_AT
GO:0033500	carbohydrate homeostasis	3	2.8301887	0.0421462	204131_S_AT, 209815_AT, 202627_S_AT, 202628_S_AT
GO:0031667	response to nutrient levels	5	4.7169811	0.0425177	39402_AT, 221841_S_AT, 209815_AT, 201041_S_AT, 205289_AT, 205290_S_AT
GO:0043067	regulation of programmed cell death	11	10.377358	0.0434225	201830_S_AT, 39402_AT, 204131_S_AT, 202036_S_AT, 219257_S_AT, 201631_S_AT, 204422_S_AT, 204363_AT, 210511_S_AT, 201010_S_AT, 201041_S_AT
GO:0051480	cytosolic calcium ion homeostasis	4	3.7735849	0.0441108	39402_AT, 204463_S_AT, 215086_AT, 218995_S_AT
GO:0010941	regulation of cell death	11	10.377358	0.0443381	201830_S_AT, 39402_AT, 204131_S_AT, 202036_S_AT, 219257_S_AT, 201631_S_AT, 204422_S_AT, 204363_AT, 210511_S_AT, 201010_S_AT, 201041_S_AT
GO:0001708	cell fate specification	3	2.8301887	0.0453075	205117_AT, 213823_AT, 204422_S_AT
GO:0030815	negative regulation of cAMP metabolic process	2	1.8867925	0.0457023	204463_S_AT, 218995_S_AT
GO:0030818	negative regulation of cAMP biosynthetic process	2	1.8867925	0.0457023	204463_S_AT, 218995_S_AT
GO:0022008	neurogenesis	9	8.490566	0.0468207	213348_AT, 208378_X_AT, 209120_AT, 204422_S_AT, 214111_AT, 210310_S_AT, 205253_AT, 213943_AT, 203131_AT, 205289_AT, 205290_S_AT
GO:0014070	response to organic cyclic substance	4	3.7735849	0.046928	39402_AT, 209815_AT, 204463_S_AT, 220109_AT

GO:0006915	apoptosis	9	8.490566	0.0471993	207574_S_AT, 201830_S_AT, 39402_AT, 204131_S_AT, 218486_AT, 201631_S_AT, 204422_S_AT, 204363_AT, 213943_AT, 209304_X_AT
GO:0008361	regulation of cell size	5	4.7169811	0.0486963	219257_S_AT, 204422_S_AT, 210511_S_AT, 204731_AT, 218995_S_AT
GO:0006916	anti-apoptosis	5	4.7169811	0.0486963	39402_AT, 202036_S_AT, 219257_S_AT, 201631_S_AT, 204363_AT
GO:0008219	cell death	10	9.4339623	0.0497829	207574_S_AT, 201830_S_AT, 39402_AT, 204131_S_AT, 218486_AT, 215350_AT, 201631_S_AT, 204422_S_AT, 204363_AT, 213943_AT, 209304_X_AT
