# Supplementary material for: Identification of a transcriptional signature for the wound healing continuum
Source: Wound Repair Regen. 2014 May 20;22(3):399–405. doi: 10.1111/wrr.12170 (PMC4230470; doi:10.1111/wrr.12170)
Supplement: Supplementary file 8 [file wrr0022-0399-SD8.pdf]

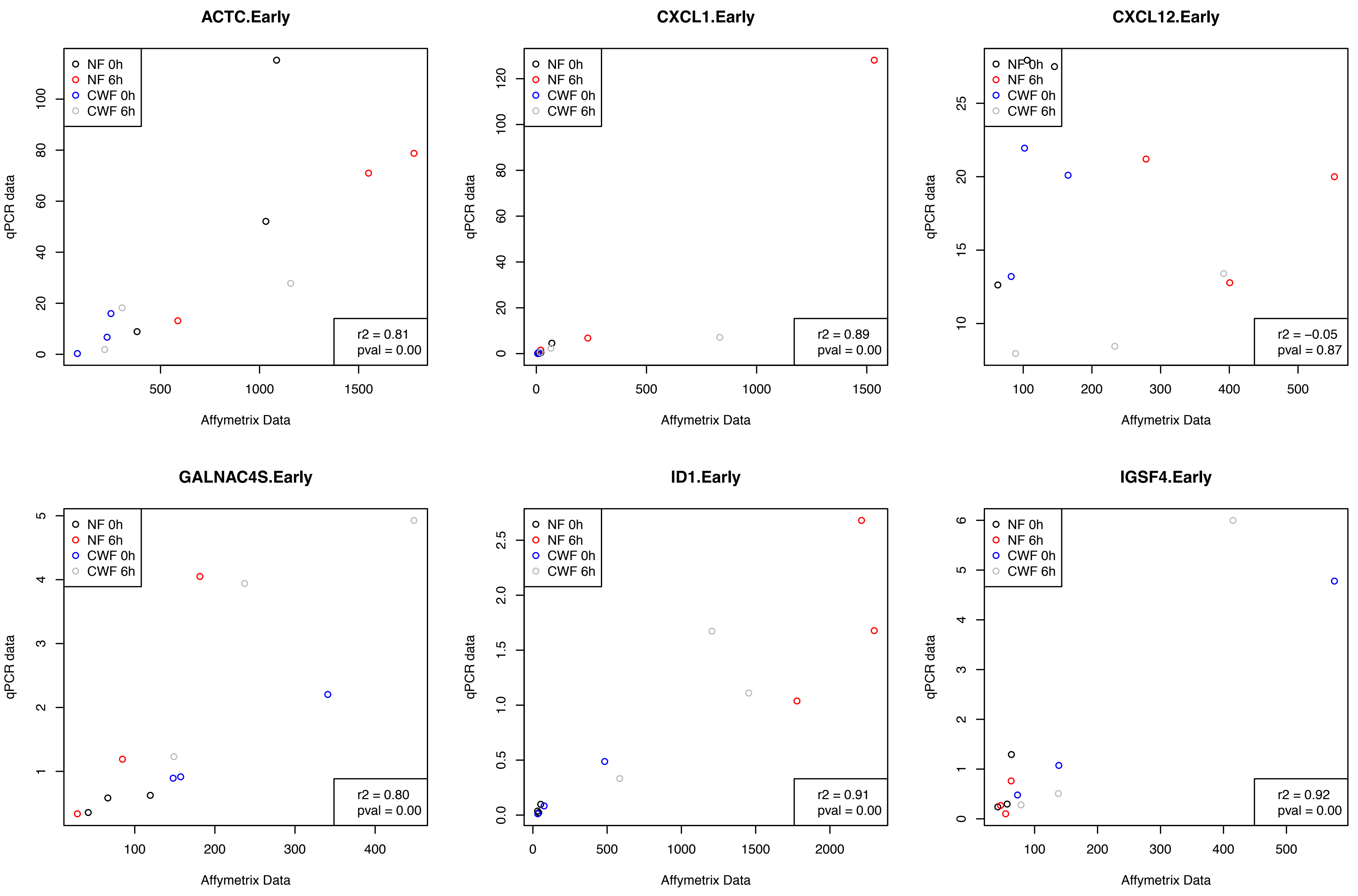
ACTC.Early
CXCL1.Early
CXCL12.Early
GALNAC4S.Early
ID1.Early
IGSF4.Early
NF 0h
NF 6h
CWF 0h
CWF 6h
qPCR data
Affymetrix Data
r2 = 0.81
pval = 0.00
r2 = 0.89
pval = 0.00
r2 = −0.05
pval = 0.87
r2 = 0.80
pval = 0.00
r2 = 0.91
pval = 0.00
r2 = 0.92
pval = 0.00

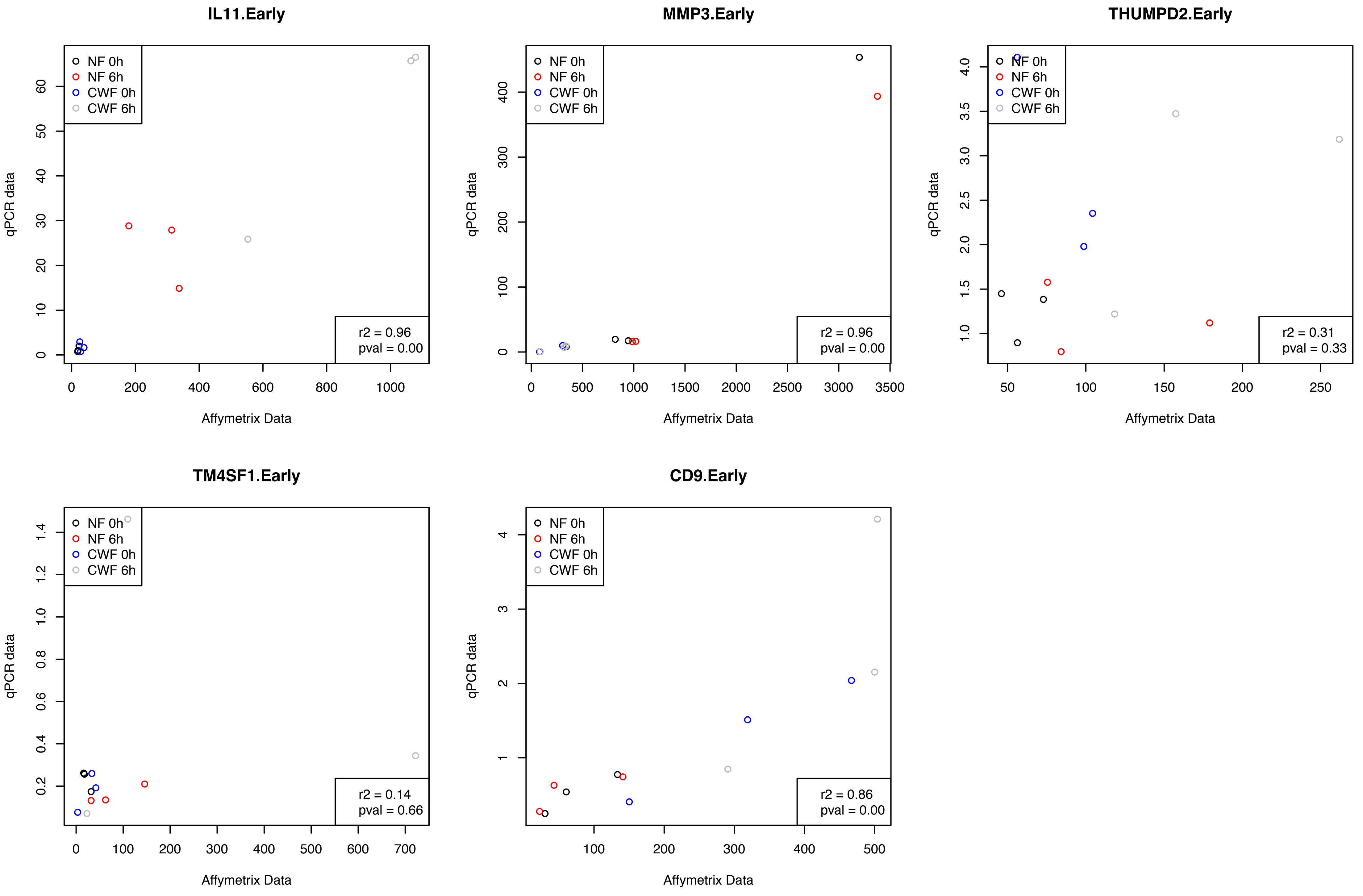
IL11.Early
NF 0h
NF 6h
CWF 0h
CWF 6h
r2 = 0.96
pval = 0.00
qPCR data
Affymetrix Data
MMP3.Early
NF 0h
NF 6h
CWF 0h
CWF 6h
r2 = 0.96
pval = 0.00
qPCR data
Affymetrix Data
THUMPD2.Early
NF 0h
NF 6h
CWF 0h
CWF 6h
r2 = 0.31
pval = 0.33
qPCR data
Affymetrix Data
TM4SF1.Early
NF 0h
NF 6h
CWF 0h
CWF 6h
r2 = 0.14
pval = 0.66
qPCR data
Affymetrix Data
CD9.Early
NF 0h
NF 6h
CWF 0h
CWF 6h
r2 = 0.86
pval = 0.00
qPCR data
Affymetrix Data
